# Green-synthesized metal nanoparticles: a promising approach for accelerated wound healing

**DOI:** 10.3389/fbioe.2025.1637589

**Published:** 2025-07-16

**Authors:** Sivakumar Singaravelu, Fezile Motsoene, Heidi Abrahamse, Sathish Sundar Dhilip Kumar

**Affiliations:** Laser Research Centre, University of Johannesburg, Johannesburg, South Africa

**Keywords:** metal nanoparticles, drug delivery, wound healing, biosensing, biomedicine, green synthesis

## Abstract

The green synthesis of metal nanoparticles (G-MNPs) in wound healing has shown a promising approach in recent decades. While chemical and physical methods have traditionally been employed for G-MNP synthesis, green synthesis methods are increasingly preferred due to their eco-friendly, safe, cost-effective, and efficient nature. These processes offer high productivity and purity without the need for high pressure, temperature, or toxic and hazardous substances, and they eliminate the need for external reducing, stabilizing, or capping agents. The green synthesis of G-MNPs can occur intra- or extracellularly and can be facilitated by various biological entities, including bacteria, fungi, yeast, algae, actinomycetes, and plant extracts. The rapid advancements in nanotechnology have been significantly propelled by the development of engineered, green-synthesized metal nanoparticles (G-MNPs). These nanoparticles have been extensively investigated for their potential applications in various biomedical fields. Their inert nature and nanoscale dimensions, which are comparable to many biological molecules, make them highly attractive in the biomedical field. Moreover, their intrinsic properties, including electronic, optical, physicochemical characteristics, and surface plasmon resonance, are highly tunable by altering parameters such as particle size, shape, environment, aspect ratio, synthesis methods, and functionalization. This tunability has facilitated their broad application in biomedicine, encompassing areas such as targeted drug delivery, biosensing, photothermal and photodynamic therapies, imaging, and the integration of multiple therapeutic modalities. This review article explores the various properties of metallic nanoparticles and their applications in the biomedical sciences while also addressing the challenges associated with their clinical translation.

## 1 Introduction

The green synthesis of metallic nanoparticles using biological pathways, particularly through living cells, is a highly efficient technique that yields a greater mass compared to other synthesis methods. Plants are rich sources of various components and biochemicals that function as both reducing and stabilizing agents in the synthesis of green nanoparticles. This method is favoured for its eco-friendliness, non-toxicity, cost-effectiveness, and enhanced stability relative to other biological, physical, and chemical methods. (Mustapha et al., 2022). Green synthesis of nanoparticles can be categorized into three main groups: extracellular, intracellular, and phytochemical methods. The use of plant extracts in nanoparticle synthesis is particularly advantageous due to the high concentration of phytochemicals present, which serve as effective reducing and stabilizing agents, facilitating the conversion of metal ions into green-synthesized metal nanoparticles (G-MNPs). (Osman et al., 2024). This approach is inexpensive and results in a higher yield compared to other methods. Green-synthesized metal and metal oxide nanoparticles are emerging as key players in the biomedical field, with applications spanning diagnostics, wound healing, tissue engineering, immunotherapy, regenerative medicine, dentistry, and biosensing platforms. Their biotoxicological, antimicrobial, antifungal, and antiviral properties have been extensively studied. (Radulescu et al., 2023). For instance, plant-mediated synthesis of copper oxide nanoparticles from various plant extracts has demonstrated diverse biological activities, including environmental remediation, photocatalysis, catalytic reduction, sensing, energy storage, and several organic transformations such as coupling, reduction, and multicomponent reactions. (Cuong et al., 2022).

The green synthesis of nanoparticles not only offers an eco-friendly, non-toxic, and cost-effective approach but also enhances the active performance of nanoparticles in removing dyes, antibiotics, and metal ions, outperforming other physical and chemical methods. (Osman et al., 2024). This method is recognized as the optimal approach for nanoparticle preparation, minimizing toxicity while increasing stability and environmental compatibility. Plants are particularly advantageous for green synthesis due to their rich phytochemical content, including phenolics, terpenoids, polysaccharides, and flavonoids, which possess oxidation–reduction capabilities. (Radulescu et al., 2023; Sampath et al., 2022). These phytochemical compounds play a crucial role in the stabilization of nanoparticles during synthesis. However, understanding the exact phytochemical composition is essential for producing stabilized nanoparticles, as plant secondary metabolites, particularly polyphenols, are significant in the green synthesis process. The green synthesis of nanoparticles is more advanced, safe, cost-effective, reproducible, and stable than other biological methods using bacteria, fungi, actinomycetes, and algae. (Ye et al., 2022; Vankudoth et al., 2022; Brar et al., 2022). Various plant parts, including roots, stems, leaves, seeds, and fruits, are involved in the synthesis of green nanoparticles due to their notable phytochemical content. The process involves washing the plant part, extracting the phytochemicals, filtering, and adding specific metal salts, followed by the extraction of nanoparticles. This method is applicable for synthesizing a wide variety of metallic nanoparticles. Green nanoparticles find applications in personal care, medicine, nano-enabled devices, food, aquaculture sciences, and agricultural products. Their eco-friendly nature makes them suitable for the industrial-scale production of green-synthesized metal nanoparticles (G-MNPs). The biosynthesis approach, involving various biological entities such as plant extracts, bacteria, yeast, seaweeds, and algae, is a crucial mechanism for avoiding harmful by-products and promoting eco-friendly and sustainable development (Parmar and Sanyal, 2022; Sikiru et al., 2022; Shafey, 2020).

Green synthesis methods are eco-friendly, non-toxic, and cost-effective, making them highly significant in the pharmaceutical industry. (Shafey, 2020). The demand for metallic nanoparticles in biology, medicine, and pharmaceuticals has surged due to their efficacy against human pathogenic microbes and their broad application in various fields. Particularly, green-synthesized metal nanoparticles (G-MNPs) are attractive in biomedical applications due to their high surface area and reactivity, which enhances production yields. (Wahab et al., 2023). These nanoparticles are classified into noble and non-noble metallic groups based on their types, and they offer an inexpensive, eco-friendly, and non-toxic approach that reduces hazardous waste accumulation. Green synthesis of metallic nanoparticles is particularly safe for biomedical and environmental applications, with significant potential as antimicrobial agents against a wide range of pathogens and in cancer treatment as nanomedicine (Maťátková et al., 2022; Alshameri and Owais, 2022).

The review emphasises the green synthesis, characterization, and application of green-synthesized metal nanoparticles (G-MNPs), such as silver, gold, iron, and copper, in antimicrobial, anticancer, and environmental remediation contexts. It highlights the superiority of green synthesis methods in producing stable, active, and environmentally friendly nanoparticles that are crucial for modern biotechnological applications. The advancement of green synthesis practices, particularly plant-based methods, offers a sustainable, safe, and cost-effective solution for the large-scale production of nanoparticles, which are increasingly in demand across multiple industries. Each one of these NPs has its specific characteristics and applications.

## 2 Green synthesis methods

Green synthesis of nanoparticles is an eco-friendly and sustainable approach that utilizes biological resources to produce nanoparticles without relying on toxic chemicals or high-energy methods (Ying et al., 2022). This strategy not only minimizes environmental impact but also yields nanoparticles with unique properties that are often difficult to achieve through conventional chemical synthesis (Singh et al., 2018). Green synthesis can be broadly categorized into plant-based synthesis, microbial synthesis, and biomolecule-assisted synthesis, each presenting its own distinct advantages and challenges (Alsaiari et al., 2023). Moreover, the summarized green synthesis procedure synthesizing various MNPs involves obtaining plant extract, mixing it with metal salt solution under specific conditions, reducing the metal particles, and filtering to obtain the target nanoscale metal (Ying et al., 2022).

While green synthesis methods have garnered considerable interest, several crucial aspects often remain unaddressed. One particularly overlooked factor is the quantitative composition of biological agents involved. Many studies tend to rely on qualitative descriptions of plant extracts or microbial cultures without adequately quantifying the active compounds that facilitate nanoparticle synthesis. This absence of standardization results in variability in the synthesis process and impacts reproducibility (Hano and Abbasi, 2022; Osman et al., 2024).

Another commonly ignored aspect is the reaction kinetics during nanoparticle formation. Monitoring the rate of reduction and nucleation is crucial for achieving uniform particle size and shape, yet this step is often omitted. Similarly, the analysis of byproducts formed during synthesis is rarely conducted, even though understanding their composition and potential environmental impact is vital for assessing the sustainability of the process (Huston et al., 2021; Alsaiari et al., 2023). The long-term stability of nanoparticles is another critical area that is frequently neglected. Factors such as storage conditions, oxidation, or aggregation over time can significantly alter nanoparticle properties, yet few studies evaluate these aspects. Additionally, the scalability and cost-effectiveness of green synthesis methods remain underexplored. While laboratory-scale processes are well-documented, the challenges of scaling up for industrial production, such as ensuring consistent quality and controlling costs, are rarely addressed (Huston et al., 2021; Samuel et al., 2022).

### 2.1 Plant-based green synthesis of nanoparticles

Plant-based synthesis is recognized as one of the most widely utilized methods to produce nanoparticles due to its simplicity, cost-effectiveness, and scalability. This approach employs aqueous extracts derived from various parts of plants, including leaves, roots, fruits, and seeds, which serve as both reducing and capping agents (Puri et al., 2024). These extracts are abundant in bioactive compounds, such as flavonoids, phenols, alkaloids, and terpenoids, that promote the reduction of metal ions to nanoparticles while simultaneously stabilising their surface. The procedure generally involves the combination of the plant extract with a metal precursor solution, carried out under meticulously controlled conditions of temperature, pH, and agitation (Vijayaraghavan and Ashokkumar, 2017; Samuel et al., 2022).

Despite its widespread application, plant-based synthesis is influenced by numerous factors that can significantly impact the properties of the resulting nanoparticles. Key parameters such as the type of plant, extraction method, and concentration of bioactive compounds play a crucial role in determining the size, shape, and stability of nanoparticles (Khan et al., 2022; Alsaiari et al., 2023). However, a commonly overlooked aspect is the standardisation of plant extracts. Variations in plant composition caused by factors like seasonality, geographical location, and cultivation practices can introduce inconsistencies in the synthesis process (Antunes Filho et al., 2023; Wang et al., 2023). These discrepancies are often disregarded, leading to challenges in reproducibility and scalability. To address this issue, it is essential to conduct rigorous characterisation and standardisation of plant extracts prior to their use in nanoparticle synthesis (Hano and Abbasi, 2022; Heinrich et al., 2022).

Plant-based synthesis demonstrates remarkable compatibility with a diverse range of metal precursors, including silver, gold, copper, and zinc salts, as well as metal oxides (Thatyana et al., 2023). This compatibility arises from the variety of phytochemicals present in plant extracts, which can effectively interact with different metal ions to facilitate their reduction and stabilisation (Shi et al., 2022). The fundamental principle of plant-based synthesis is rooted in the redox chemistry of the phytochemicals found in plant extracts (Thatyana et al., 2023). These compounds serve as reducing agents by donating electrons to metal ions, thereby reducing them to their zero-valent nanoparticle form. Additionally, certain bioactive molecules act as capping agents, creating a stabilising layer on the nanoparticle surface to prevent aggregation. This dual role of phytochemicals—as both reducers and stabilisers are essential for the success of the synthesis process (Javed et al., 2022; Villagrán et al., 2024). The size, shape, and stability of the nanoparticles are influenced by the relative concentrations of the reducing and capping agents, as well as the reaction conditions, including pH, temperature, and precursor concentration (Gulcin and Alwasel, 2022).

The nanoparticle synthesis process is initiated with the preparation of a plant extract by boiling or macerating plant material in water or another solvent (Alabdallah and Hasan, 2021). This initial step facilitates the extraction of bioactive compounds that are instrumental in both the reduction and stabilization of nanoparticles. Following the filtration to eliminate solid residues, the resulting clear extract is combined with a metal precursor solution, such as silver nitrate for the synthesis of silver nanoparticles or chloroauric acid for gold nanoparticles (Bharadwaj et al., 2021; Giri et al., 2022). The reaction mixture is then maintained under carefully controlled conditions of temperature and pH, which are optimised according to the specific plant extract and metal precursor used. During the reaction, the bioactive compounds in the extract reduce the metal ions to their zero-valent state, facilitating the nucleation and growth of nanoparticles (Khan et al., 2022). Concurrently, other constituent functions as capping agents, stabilising the nanoparticles and preventing aggregation. The final product is purified through centrifugation or filtration to remove unreacted precursors and impurities (Samuel et al., 2022).

The biomedical application of plant-based synthesis presents numerous advantages, making it a preferred method for nanoparticle production. This method is environmentally sustainable, as it does not necessitate the use of hazardous chemicals or energy-intensive processes (Singh et al., 2023). The incorporation of natural plant materials renders the technique not only cost-effective but also widely accessible. Moreover, nanoparticles generated via this process frequently demonstrate improved biocompatibility, attributable to the presence of bioorganic capping agents, thereby enhancing their suitability for biomedical applications. Additionally, the scalability of this methodology facilitates its application in industrial-scale production, provided that appropriate optimisations are implemented (Begum and Jayawardana, 2023).

Despite these advantages, there are several limitations to this method. A major challenge is variability in plant composition due to environmental factors such as seasonal changes, geographical location, and cultivation practice (Kulkarni et al., 2023). These variations can lead to inconsistencies in the synthesis process, affecting nanoparticle size, shape, and stability. Moreover, the lack of standardised protocols for extract preparation and reaction conditions can hinder reproducibility. Yield and purity may also be lower compared to conventional chemical methods, and the presence of organic residues from the plant extract can complicate downstream applications (Hano and Abbasi, 2022; Ying et al., 2022).

### 2.2 Microbial-based green synthesis of nanoparticles

Microbial-based green synthesis of GMNPs uses the metabolic activity of microorganisms such as bacteria, fungi, algae or yeast to reduce metal ions and stabilise nanoparticles. These organisms secrete enzymes, proteins, and metabolites capable of acting as reducing and capping agents (Ghosh et al., 2021; Ali et al., 2024). This method is particularly advantageous for its specificity and the ability to produce nanoparticles with well-defined shapes and sizes. Microbial synthesis is also considered environmentally friendly, as it typically occurs under mild reaction conditions and without the use of hazardous chemicals (Iravani, 2014; Sudheer et al., 2022).

Key factors influencing microbial synthesis include the choice of microorganisms, the composition of the culture medium, and the environmental conditions, such as pH, temperature, and nutrient availability (Guilger-Casagrande et al., 2021; Ali et al., 2024). The pH of the medium is a critical factor influencing the size, shape, and stability of nanoparticles (NPs). Microorganisms exhibit various responses to different pH levels, which affect the redox potential and enzymatic activity that are integral to NP synthesis. Furthermore, temperature serves as another essential parameter, significantly impacting reaction rates and the kinetics associated with NP formation. Additionally, the concentration of precursor compounds within the growth medium is a fundamental determinant of both NP yield and size (Priyadarshini et al., 2021; Rami et al., 2024). However, significant challenges arise from the lack of standardised protocols for microbial cultivation and nanoparticle recovery. The metabolic activity of microorganisms can vary widely depending on the strain, growth conditions, and age of the culture. These variations are often not fully characterised, leading to inconsistencies in nanoparticle synthesis. Additionally, the purification of nanoparticles from microbial biomass can be complex and time-consuming, a step that is frequently underestimated in the overall process (Grasso et al., 2019; Kapinusova et al., 2023).

GMNPs microbial synthesis supports a wide range of metal precursors, including iron, silver, gold, copper, and zinc salts, as well as metal oxides. Its compatibility stems from the metabolic versatility of microorganisms, which interact with metal ions through enzymatic and non-enzymatic pathways (Pulingam et al., 2022; Gonfa et al., 2023). The selection of compatible micro-organism plays a crucial role in determining the efficiency and characteristics of green-synthesized metal nanoparticles (G-MNPs). Bacteria like *Pseudomonas aeruginosa* and fungi such as *Aspergillus flavus* are commonly used due to their strong nanoparticle-producing capabilities. However, optimizing factors like pH, temperature, and nutrient composition is essential to enhance yield and quality (Ahmad et al., 2019b; Kumar et al., 2022; Noman et al., 2023). The synthesis process can occur intracellularly and extracellularly, where metal ions penetrate microbial cells and are reduced by enzymes, or extracellularly, where secreted biomolecules facilitate reduction and stabilization. Microbial redox reactions play a key role, with enzymes like nitrate reductase converting metal ions into nanoparticles while proteins and polysaccharides stabilize them. This process ensures nanoparticles form in specific shapes, such as spheres, rods, or triangles, and sizes ranging from a few to tens of nanometres (Markus et al., 2016; Mahdi et al., 2021; El-Bendary et al., 2021; Álvarez-Chimal and Arenas-Alatorre, 2023).

Microbial synthesis offers numerous advantages, making it a compelling method for green nanoparticle production. It is highly eco-friendly, as it utilizes renewable biological resources and operates under mild reaction conditions (Alsaiari et al., 2023; Álvarez-Chimal and Arenas-Alatorre, 2023). The method is cost-effective, given the low cost of microbial cultivation and the elimination of expensive chemicals. Additionally, the nanoparticles synthesized through this approach often exhibit enhanced biocompatibility due to the presence of biomolecular coatings, making them suitable for biomedical applications such as drug delivery and imaging and cell signalling. Furthermore, microbial synthesis provides an avenue for large-scale production, particularly when optimized for industrial applications (Lahiri et al., 2021; Alsaiari et al., 2023; Kiarashi et al., 2024). However, the method also has several limitations. The growth and metabolic activity of microorganisms can be sensitive to environmental factors, making the process less predictable and reproducible compared to chemical methods. Intracellular synthesis poses challenges in isolating nanoparticles from the cell matrix, which can add complexity to the purification process. The variability in microbial strains and culture conditions can lead to inconsistencies in nanoparticle size, shape, and yield. Moreover, microbial synthesis is slightly slower compared to other methods, which may limit its scalability without significant optimisation. (Álvarez-Chimal and Arenas-Alatorre, 2023; Kiarashi et al., 2024).

### 2.3 Biological reduction and surface functionalization in gren synthesis of meal nanoparticles

The biosynthesis of metal nanoparticles (MNPs) using plant extracts and microorganisms presents a clean, cost effective, and environmentally friendly alternative to conventional chemical and physical methods. At the heart of this process lies a complex cascade of biochemical events involving the reduction of metal ions and stabilization of nanoparticles via surface functionalization. This section elaborates on the molecular mechanisms underpinning these processes, supported by literature (Song and Kim, 2009).

#### 2.3.1 Biochemical reduction mechanisms in plant-based synthesis

The green synthesis of metal nanoparticles using plant extracts primarily relies on the rich diversity of secondary metabolites present in the plant tissues. These bioactive compounds such as polyphenols, flavonoids (e.g., quercetin, catechin, kaempferol), terpenoids, tannins, reducing sugars, and ascorbic acid serve as natural reducing and stabilizing agents. When a metal salt (e.g., AgNO_3_, HAuCl_4_, ZnSo_4_) is introduced into the plant extract, these phytochemicals interact with the metal ions (Ag^+^, Au^3+^, Zn^2+^) and reduce them to their elemental metallic forms (Ag^0^, Au^0^, Zn^0^). The redox reactions typically involve the oxidation of hydroxyl and carboxyl groups present in these biomolecules. For instance, polyphenols such as catechol can donate electrons to reduce Ag^+ ^to Ag^0^ while being oxidized to quinones in the process. A representative reaction is,

$${\text{Ag}}^{+}+\text{Polyphenol }\left(\text{catechol}\right)\to \;{\text{Ag}}^{0}+\text{Oxidized polyphenol}$$

This electron transfer mechanism plays a central role in nanoparticle formation. Additionally, molecules like ascorbic acid contribute significantly by offering strong reduction power while enhancing the antioxidant stability of the synthesis environment. (Song and Kim, 2009). Following the reduction step, the formation of nanoparticles proceeds through a nucleation process wherein reduced metal atoms aggregate into small clusters. Key factors influencing this stage include the pH of the extract, which affects the ionization of functional groups the concentration of both the metal precursor and the phytochemicals, as well as reaction parameters such as temperature and time. These factors together control whether the nanoparticles develop into spherical, triangular, rod-shaped, or other anisotropic forms. Specific phytochemicals can selectively adsorb onto certain crystallographic facets of the nanoparticles, thereby guiding their growth pattern and contributing to shape-controlled synthesis.

#### 2.3.2 Surface functionalization: Capping and stabilization

Nanoparticles synthesized through green methods demonstrate exceptional colloidal stability, largely attributed to *in situ* surface functionalization by various bio-organic molecules present in plant extracts. This surface modification process, also known as capping, involves the adsorption or binding of phytoconstituents such as proteins, tannins, phenolics, amino acids, and sugars onto the surface of the newly formed nanoparticles. These naturally occurring compounds act as stabilizing agents, effectively preventing the aggregation of nanoparticles by providing steric hindrance and electrostatic repulsion. Additionally, they improve the solubility and dispersibility of nanoparticles in aqueous and biological environment. Among the various capping agents found in plant extracts, proteins play a crucial role by binding to nanoparticle surfaces through amino and carboxyl functional groups, forming a protective corona. Sugars and polysaccharides, such as those derived from aloe vera and gum Arabic, contribute to stabilization through steric hindrance, creating a physical barrier that inhibits particle aggregation. Furthermore, phenolic compounds and tannins interact with nanoparticles via hydrogen bonding and π–π stacking interactions, forming, non-covalent interactions that reinforce particle stability. These capping agents not only stabilize the nanoparticles but also enhance their biocompatibility, making them ideal candidates for a variety of biomedical applications including targeted drug delivery, diagnostic imaging, and photothermal therapy. A well-documented example involves the use of Ocimum sanctum (holy basil) leaf extract, in which flavonoids and terpenoids simultaneously reduce Au^3+^ ions to elemental gold (Au^0^) and act as natural capping agents. This dual functionality yields highly uniform and stable gold nanoparticles, showcasing the intrinsic advantage of plant-based synthesis (Ankamwar et al., 2005).

#### 2.3.3 Microorganisms-mediated reduction and functionalization

In addition to plant-based systems, microorganisms such as fungi and bacteria serve as efficient biological agents for the green synthesis of metal nanoparticles. These microbes facilitate both the reduction of metal ions and the surface functionalization of the resulting nanoparticles through the action of various intracellular and extracellular enzymes and metabolites. This biogenic approach offers an eco-friendly and scalable alternative for nanoparticle synthesis (Bahrulolum et al., 2021).

##### 2.3.3.1 Enzymatic reduction

One of the primary mechanisms by which microbes reduce metal ions involves enzyme-mediated redox reactions. Enzymes such as nitrate reductase, hydrogenase, and sulfur reductase play significant roles in the detoxification of metal ions by converting them into their elemental nanoparticle forms. For example, nitrate reductase utilizes NADH as an electron donor to reduce metal ions like Ag^+^ to Ag^0^. A well-known case involves the fungus *Fusarium oxysporum*, which secretes nitrate reductase into the extracellular environment, leading to the efficient biosynthesis of silver nanoparticles. These enzymes not only reduce metal ions but also influence the kinetics and morphology of nanoparticle formation (Campaña et al., 2023).

##### 2.3.3.2 Protein capping

Once the metal ions are reduced, stabilization of the resulting nanoparticles is achieved through protein-mediated capping. Microbial cells release extracellular proteins that adhere to the nanoparticle surface via functional groups such as thiol (-SH), amine (-NH_2_) and carboxyl (-COOH). These biomolecular ligands act as natural capping agents, forming a protective layer around the nanoparticles that prevents their aggregation and promotes uniform dispersion. In bacteria such as *pseudomonas aeruginosa*, intracellular synthesis of gold nanoparticles is accompanied by the binding of cellular peptides and proteins, forming a bio-organic shell that enhances nanoparticle stability and biocompatibility. This protein mediated surface functionalization is critical for ensuring the long-term stability and functional integration of biosynthesized nanoparticles in various applications (Bhainsa and D’souza, 2006).

#### 2.3.4 Synergistic actions and factors influencing surface functionalization

In green synthesis, both the reduction of metal ions and their surface functionalization are often mediated by the same or closely related biomolecular species, such as polyphenols, proteins, and sugars. This synergistic interplay ensures that nanoparticles are not formed but are also stabilized and functionalized simultaneously. Such dual functionality is a key advantage of green synthetic routes, as it contributes to the development of nanoparticles that are stable, biocompatible, and readily adaptable for various downstream applications in biomedicine, agriculture, and environmental remediation. Several factors influence the efficiency and outcome of surface functionalization. The molecular weight of the capping agents, such as proteins *versus* smaller molecules like sugars, affects he steric stabilization and the density of surface coverage (Iravani, 2011). The iconic strength and pH of the medium play a critical role by altering the ionization states of functional groups and influencing electrostatic interactions between the capping molecules and the nanoparticle surface. Additionally, temperature and light exposure can modulate reaction kinetics and potentially activate or deactivate certain phytoconstituents involved in capping. The polarity of the solvent and the chemical composition of the plant or microbial extract also dictate the availability and orientation of functional groups, thus impacting the uniformity and stability of the final nanoparticle formulation (Anil Kumar et al., 2007). In conclusion, the green synthesis of metal nanoparticles is governed by a dynamic and interconnected series of events involving both biochemical reduction and surface functionalization. Plants and microorganisms act as natural nano-factories, facilitating the co-friendly reduction of metal ions and concurrently passivating and functionalizing the nanoparticles. This comprehensive mechanism provides a robust foundation for producing safe, scalable, and application-specific nanomaterials, particularly in areas such as targeted drug delivery, diagnostics, and theranostic systems (Mittal et al., 2013).

## 3 The potential role and functions of green-synthesized metal nanoparticles (G-MNPs)

### 3.1 Eco-friendly synthesis

In the green synthesis of nanoparticles, naturally occurring elements such as microbes and plant extracts are used to create environmentally safe components that serve as reducing and stabilizing agents. This method greatly reduces the need for dangerous chemicals that are usually used in traditional synthesis procedures. Compared to alternative techniques, the biological manufacturing of green nanoparticles within live cells is more effective and produces larger quantities (Ying et al., 2022). Numerous components and biochemicals that can function as stabilizers and reducers during the creation of nanoparticles can be found in abundance in plants. Green synthesis approaches are distinguished from conventional biological, physical, and chemical procedures by their greater stability, non-toxicity, affordability, and environmental friendliness. (Mustapha et al., 2022).

Green nanoparticles can be synthesized using three main techniques: extracellular, intracellular, and phytochemical-mediated. The phytochemical elements found in abundance in plant extracts serve as both stabilizing and reducing agents, making it easier for metal ions to be reduced to G-MNPs. Higher nanoparticle yields are produced by this method, which is also economical (Venkataraman, 2022).

### 3.2 Biocompatibility

**FIGURE 1:**
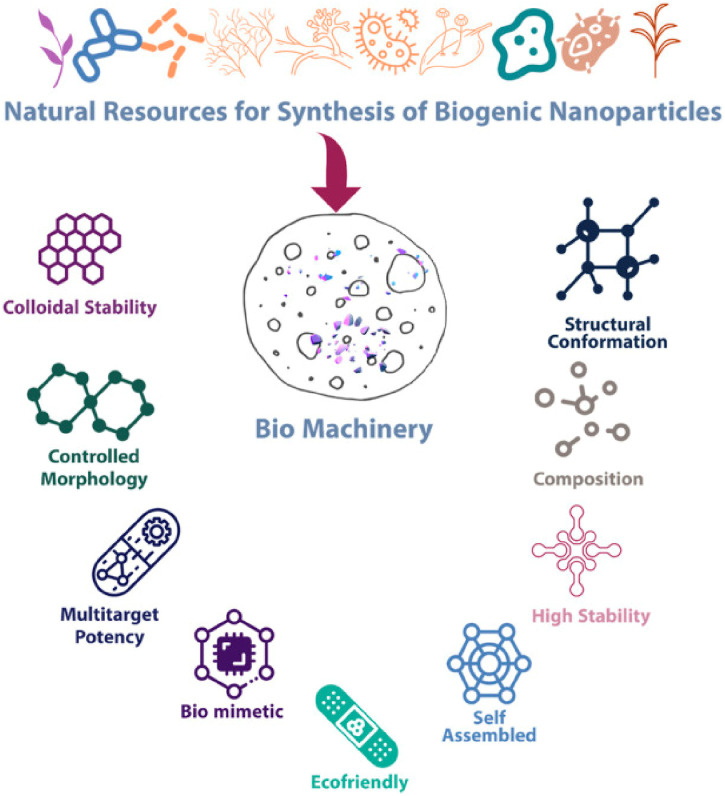
Salient features and properties of biogenic nanoparticles (Kulkarni et al., 2023).

Green synthesis, which frequently uses biological entities like plant extracts or microorganisms, produces nanoparticles with intrinsic biocompatibility. This technique makes them appropriate for a range of biomedical uses, such as treatment, imaging, and medication delivery. As a result, eco-friendly methods that make use of biopolymers, plant extracts, and biomolecules have gained importance (Ahmed et al., 2016b). In addition to acting as capping, reducing, and shape-modulating agents, these materials are accessible and biocompatible, making them perfect reagents. The many benefits and crucial significance of biogenic synthesis are illustrated in Figure 1. Clean analytical methods, environmentally friendly analytical chemistry, and green analytical chemistry are all heavily reliant on green chemistry, which uses chemicals to reduce pollution. The manufacturing of nanoparticles using green synthesis is especially appealing because of its environmental safety, inertness, and biocompatibility (Scala et al., 2022).

### 3.3 Narrow size distribution

Green synthesis approaches often facilitate the production of nanoparticles with a narrow size distribution, a critical parameter for ensuring uniform physicochemical properties and reproducible performance in various applications. Microorganisms play a pivotal role in biogenic nanoparticle synthesis through both direct and indirect mechanisms. However, microbial-mediated synthesis is often characterized by slow reaction kinetics, posing challenges in controlling the heterogeneity of microbial species involved. Furthermore, nanoparticles synthesized via biological routes frequently exhibit variations in size distribution, necessitating specialized expertise during the manufacturing process. The requirement for skilled personnel can significantly elevate the costs associated with large-scale production and industrial translation (Saif et al., 2016).

### 3.4 Surface functionalization

**FIGURE 2:**
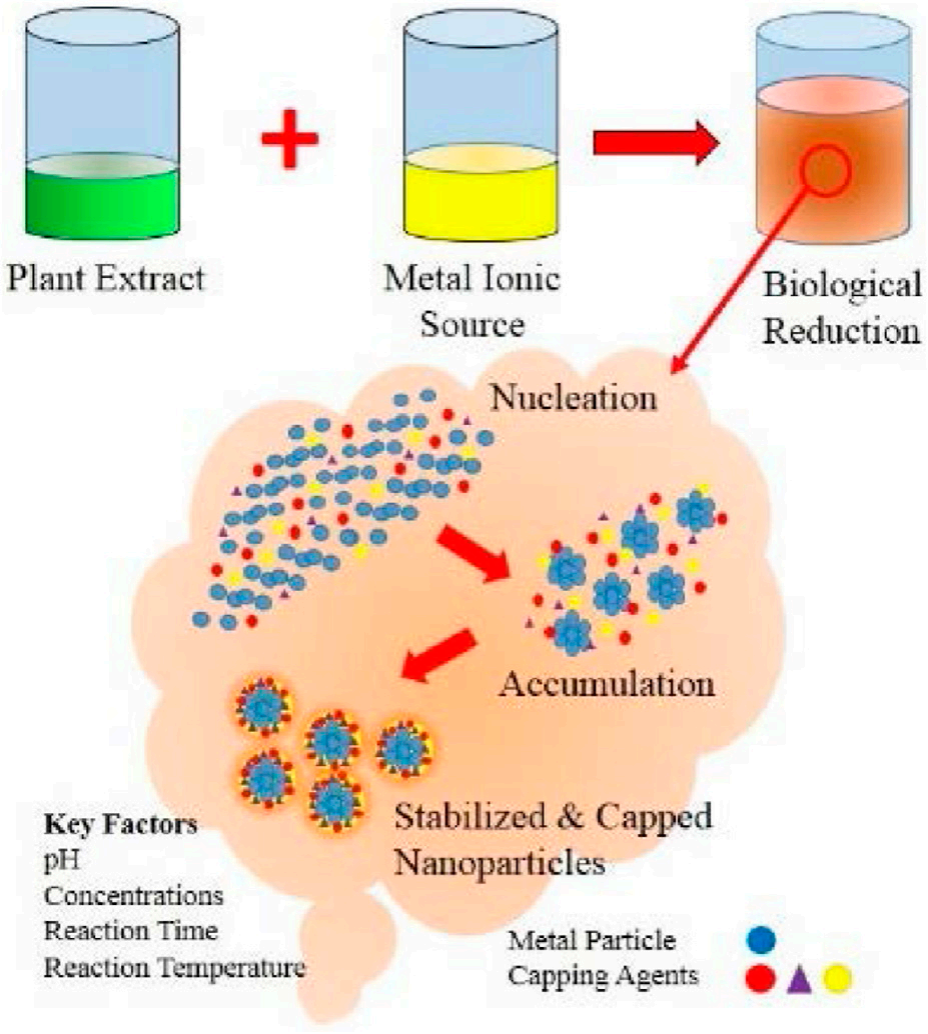
Biological synthesis of nanoparticles using plant extracts (Shah et al., 2015).

The surface of green-synthesized nanoparticles can be effectively functionalized by modulating the biological components utilized during the synthesis process. This functionalization enhances their stability, biocompatibility, and specificity for targeted applications. Surface modification of nanoparticles can be accomplished through two principal approaches: (i) *in situ* functionalization, a one-step process wherein synthesis and surface modification occur concurrently, and (ii) post-synthesis modification, a sequential approach involving nanoparticle synthesis followed by subsequent surface modification. The physicochemical properties of the coating materials and the specific application requirements dictate the choice of coating strategy. Typically, nanoparticle surface functionalization involves ligand attachment, ligand exchange, or encapsulation, each tailored to optimize performance in diverse biomedical and technological applications (Thanh and Green, 2010). The surface functionalization of green-synthesized metal nanoparticles is illustrated in Figure 2.

### 3.5 Enhanced stability

Green-synthesized nanoparticles demonstrate enhanced stability due to the presence of natural stabilizing agents, which contribute to extended shelf life and consistent performance. Chemical vapor deposition (CVD) is a widely employed technique for depositing thin films onto surfaces using vapor-phase precursors, enabling the production of high-quality, uniform, and durable nanoparticles suitable for various applications (Baig et al., 2021). Green synthesis methodologies utilize bioactive agents derived from plant extracts, microorganisms, and biowastes to fabricate G-MNPs, presenting an eco-friendly, cost-effective, and scalable alternative with superior stability and non-toxic byproducts (Malhotra and Alghuthaymi, 2022). Within biological systems, NADH-dependent reductases facilitate electron transfer from metal ions to their elemental states, driving nanoparticle synthesis and stabilization through interactions with proteins and amino acids (Mohd Yusof et al., 2019).

Gold nanoparticles (AuNPs) are renowned for their unique optical properties, facile synthesis, and exceptional chemical stability, making them highly advantageous for applications in cancer therapy, bioimaging, biosensing, and targeted drug delivery (Sun et al., 2021). Their ability to facilitate controlled and site-specific drug release further enhances their therapeutic potential. Similarly, silver nanoparticles (AgNPs), zinc oxide nanoparticles (ZnONPs), and copper nanoparticles (CuNPs) exhibit distinct functionalities, including tumor-targeting capabilities, selective cytotoxicity toward cancer cells, and antimicrobial efficacy, respectively. The integration of nanoparticles into defence materials significantly enhances mechanical strength, thermal stability, and electrical conductivity, thereby improving overall performance and durability (Siddique and Chow, 2020; Anjum et al., 2021; Yuan et al., 2018). In energy storage applications, nanoparticles play a pivotal role in augmenting the efficiency and performance of batteries and fuel cells. As cathode materials in batteries, they contribute to increased energy density, enhanced rate capability, and improved cycling stability. In supercapacitors, nanoparticles effectively increase the specific surface area of electrode materials, leading to enhanced capacitance. Collectively, these advancements in nanotechnology substantially improve the performance, efficiency, and safety of energy storage systems utilized in defence applications (Morsi et al., 2022).

### 3.6 Tunable properties

Green-synthesized nanoparticles (NPs) offer tunable physicochemical properties, including size, morphology, and surface chemistry, which can be precisely modulated during synthesis to meet specific application requirements. This adaptability makes them highly suitable for catalytic processes, sensing technologies, and environmental remediation. Green-synthesized metal nanoparticles (G-MNPs), in particular, exhibit exceptional catalytic efficiency, enabling chemical transformations at lower temperatures. For instance, platinum nanoparticles (PtNPs) are extensively utilized in fuel cell reactions, hydrogenation, and oxidation processes (Bhavani et al., 2021; Lara and Philippot, 2014); palladium nanoparticles (PdNPs) play a crucial role in hydrogenation and cross-coupling reactions (Pérez-Lorenzo, 2012); iron nanoparticles (FeNPs) facilitate hydrolysis and oxygen reduction reactions (Jiang and Xu, 2011); while nickel nanoparticles (NiNPs) contribute to hydrogenation and hydrolysis processes (Salem and Fouda, 2021).

Iron nanoparticles (FeNPs), typically ranging from 1 to 100 nm in size, find applications across diverse fields, including catalysis, targeted drug delivery, biosensing, energy storage, solar cell development, water purification, and as contrast agents in magnetic resonance imaging (MRI) (Khan et al., 2019). Mechanical milling techniques are commonly employed to downsize bulk materials into nanoscale structures, yielding reinforced aluminum alloys, wear-resistant coatings, and advanced nanocomposites with enhanced mechanical properties (Zhuang and Gentry, 2011; Jamkhande et al., 2019). Nanoparticles also play a critical role in biofuel production and environmental remediation. Platinum nanoparticles (PtNPs) have demonstrated efficacy in biomass-to-fuel conversion and in sensing applications, particularly for detecting Mercury(I) ions (Hg) in aqueous environments (Lam and Luong, 2014). While the application of green-synthesized metal nanoparticles (G-MNPs) holds significant promise, their development presents both challenges and opportunities for future advancements in electronics, energy storage, catalysis, and biomedical sciences (Kora and Rastogi, 2018).

### 3.7 Antimicrobial activity

Green-synthesized nanoparticles inherently exhibit potent antimicrobial properties, making them highly effective against a broad spectrum of microorganisms. This attribute is particularly valuable in applications such as antimicrobial coatings, food packaging, and water purification (Nandhini et al., 2023). Silver nanoparticles (AgNPs) are widely recognized for their broad-spectrum antibacterial efficacy and minimal cytotoxicity toward mammalian cells. As a result, they are extensively employed in wound dressings, antimicrobial gels, orthopedic implants, medical catheters, surgical instruments, implants, contact lens coatings, and additive manufacturing technologies (3D and 4D printing) (Pangli et al., 2021; Varaprasad et al., 2022). AgNPs synthesized using plant, fungal, and bacterial extracts exhibit significant antimicrobial potency (Ahmad et al., 2019a). For instance, AgNPs derived from Coriolus versicolor and Boletus edulis demonstrate strong antibacterial activity against both Gram-positive bacteria (*Staphylococcus aureus*, *Enterococcus faecalis*) and Gram-negative bacteria (*Pseudomonas aeruginosa*, *Klebsiella pneumoniae*). Furthermore, these nanoparticles enhance the antibacterial efficacy of chloramphenicol against methicillin-resistant *S. aureus* (MRSA) (Kaplan et al., 2021).

Zinc oxide (ZnO) nanoparticles exert antimicrobial effects by generating reactive oxygen species (ROS) upon exposure to light, effectively inhibiting microbial growth. ZnO nanoparticles are characterized by their biocompatibility, non-toxic nature, cost-effectiveness, environmental sustainability, and optical transparency, making them ideal for advanced biomedical applications (Kaushik et al., 2019). Green synthesis methodologies further enhance the functionality of ZnO nanoparticles by optimizing their particle size, photocatalytic activity, degradation efficiency, biocompatibility, antioxidant properties, and antibacterial potential, particularly in wound healing applications. Their high surface area and superior adsorption properties contribute to their enhanced antimicrobial efficacy (Faisal et al., 2021). We summarised the mechanism of wound healing and bactericidal activities of different G-MNPs in the below-mentioned Table 1.

**TABLE 1:** Comparison between green-synthesized and chemically synthesized nanoparticles in wound healing models.

Parameter	Green-synthesized nanoparticles (G-MNPs)	Chemically synthesized nanoparticles (C-MNPs)
Synthesis Approach	Uses biological agents (plants, fungi, bacteria) as eco-friendly reducing/stabilizing agents	Involves chemical reducing agents like NaBH_4_ or citrate, often toxic (Mittal et al., 2013)
Cytotoxicity	Lower; exhibits good compatibility with skin cells (fibroblasts, keratinocytes)	Higher; may induce ROS or apoptosis due to chemical residues (Bukhari et al., 2021)
Cellular Response (*In Vitro*)	Promotes fibroblast proliferation, migration, and collagen synthesis	Moderate or variable response; less stimulation of regeneration pathways (Ahmed et al., 2016b)
Wound Closure Rate (*In Vivo*)	Accelerated wound closure, angiogenesis, and re-epithelialization observed in murine and rat models	Slower healing in comparison, often with prolonged inflammation (Balakumaran et al., 2016)
Histological Outcome	Improved tissue remodeling with aligned collagen, fewer inflammatory cells	Less organized matrix deposition; moderate inflammatory infiltrates (Shahzadi et al., 2025)
Anti-inflammatory/Antioxidant Properties	Strong ROS scavenging; reduces IL-6, TNF-α expression	Often absent or limited; may exacerbate oxidative stress (Slavin et al., 2017)
Antimicrobial Activity	Strong inhibition of pathogens and biofilm due to phytochemical synergy	Effective, but may require higher concentration to match G-MNPs(Mule, 2024)
Environmental Impact & Cost	Low-cost, sustainable, and suitable for large-scale production	Higher cost, generates hazardous waste (Iravani, 2011)

### 3.8 Biodegradability

The green synthesis of green-synthesized metal nanoparticles (G-MNPs) (NPs) leverages biological entities such as plants, bacteria, fungi, and algae to facilitate the bio-reduction of metal ions into nanoparticles. This environmentally sustainable approach yields biocompatible and biodegradable nanoparticles, making them highly suitable for various biomedical applications, particularly in wound healing. A key characteristic of G-MNPs is their enhanced biodegradability, primarily conferred by natural capping agents derived from biological sources. These capping agents, consisting of proteins, polysaccharides, and other biopolymers, play a pivotal role in regulating the gradual degradation of nanoparticles within biological systems. This controlled degradation enables the sustained release of metal ions, which actively contribute to tissue regeneration and the overall wound healing process (Radulescu et al., 2023).

### 3.9 Wound healing properties

Wound healing is a complex biological process that involves multiple phases, including hemostasis, inflammation, proliferation, and tissue remodeling. G-MNPs, such as Silver (AGNPs0, Gold (AUNPs), and Zinc Oxide (ZnO NPs), have shown significant potential in enhancing wound healing due to their antibacterial, anti-inflammatory, pro-angiogenic, and collagen-promoting properties.

One of the primary challenges in wound healing is infection, which can delay the process and lead to complications. G-MNPs exhibit strong antibacterial activity through various mechanisms. They disrupt bacterial cell membranes, causing increased permeability and structural damage, ultimately leading to cell death. Additionally. These nanoparticles induce the generation of reactive oxygen species (ROs), which contribute to oxidative stress, resulting in lipid peroxidation, protein degradation, and DNA fragmentation within bacterial cells. Furthermore, G-MNPs interfere with bacterial DNA replication, and protein synthesis, preventing microbial proliferation. By effectively eliminating infections at the wound site, these nanoparticles create a sterile environment, reducing the risk of complications and promoting faster healing (Shenashen et al., 2014; Jeyaraj et al., 2019). Inflammation plays a crucial role in wound healing; however, excessive inflammation can hinder tissue repair and lead to chronic wounds. G-MNPs help regulate inflammation by suppressing pro-inflammatory cytokines such as TNF-α, IL-6, and IL-1β, which are association with prolonged inflammation. At the same time, they enhance the expression of anti-inflammatory cytokines like IL-10, thereby ensuring a balanced immune response. Moreover, these nanoparticles reduce oxidative stress by neutralizing free radicals, minimizing cellular damage at the wound site. By modulating inflammation, G-MNPs create a favorable environment for tissue regeneration, leading to quicker and more efficient wound closure (Shenashen et al., 2014).

Angiogenesis, the formation of new blood vessels, is essential for supplying oxygen and nutrients to the wound site, facilitating tissue regeneration. Certain G-MNPs, particularly AUNPs and ZnO NPs, stimulate angiogenesis by upregulating vascular endothelial growth factor (VEGF) expression, which enhances new capillary formation. These nanoparticles also improve endothelial cell proliferation and migration, further supporting blood vessel development. Enhanced angiogenesis ensures an adequate oxygen and nutrient supply to the regenerating tissue, thereby accelerating wound closure, especially in chronic or non-healing wounds (Rajendran et al., 2018). Collagen is a fundamental component of the extracellular matrix (ECM), providing structural integrity and tensile strength to healed tissues. G-MNPs promote collagen synthesis by stimulating fibroblast proliferation and migration, which are essential for ECM deposition. Additionally, these nanoparticles regulate the expression of collagen-producing genes such as COL1 and COL3 while enhancing the activity of transforming growth factor-beta (TGF- β), a key factor in tissue remodeling and fibrosis. Increase collagen deposition leads to stronger, more resilient wound tissue, reducing the risk of reinjury and improving the overall healing outcome (Atala et al., 2010).

#### 3.9.1 Different types of G-MNPs in wound healing

Green-synthesized AgNPs are widely recognized for their potent antimicrobial properties, which help reduce the microbial load at the wound site and prevent infections. In addition to their antibacterial effects. AgNPs enhance fibroblast migration and proliferation, two critical processes for tissue repair. They also exhibit anti-inflammatory properties, helping to regulate the immune response and prevent excessive inflammation. Furthermore, AgNPs accelerate re-epithelization, the process by which new skin layers form over the wound, ultimately leading to faster wound closure and tissue regeneration. (Shenashen et al., 2014). Biocompatible and biodegradable, AuNPs synthesized via plant-based green synthesis techniques plays a significant role in wound healing. These nanoparticles promote cell proliferation and migration, particularly of keratinocyte and fibroblasts, which are essential for tissue repair. Additionally, AuNPs help mitigate oxidative stress at the wound site by neutralizing free radicals, reducing cellular damage, and improving overall tissue regeneration. Another key benefit of AuNPs is their ability to stimulate angiogenesis, ensuring an adequate blood supply to the wound and enhancing the healing process (Jeyaraj et al., 2019).

Green-synthesized ZnO NPs have gained attention due to their multifunctional properties in wound healing. These nanoparticles possess strong antibacterial effects, effectively eliminating wound pathogens and reducing the risk of infections. Their anti-inflammatory properties further contribute to the healing process by modulating immune responses and preventing excessive inflammation. Moreover, ZnO NPs stimulate fibroblast and keratinocyte activity, leading to enhanced collagen synthesis and faster wound closure. By promoting both re-epithelization and extracellular matrix formation, ZnO NPs support efficient wound healing and tissue repair (Jeyaraj et al., 2019). PtNPs are distinguished by their exceptional physicochemical properties, including corrosion resistance, high surface area, and chemical inertness. These nanoparticles exhibit antibacterial and antitumor properties and have demonstrated potential applications in oxidative stress reduction, cancer cell detection, and neurodegenerative disease treatment, including Parkinson’s disease. Green-synthesized PtNPs, produced using naturally occurring reducing biopolymers, are biodegradable, biocompatible, highly stable, and osteoconductive, making them promising candidates for regenerative medicine applications (Gong et al., 2015; Trivedi et al., 2022). MgO NPs are highly valued for their non-toxicity, biocompatibility, and exceptional stability under extreme conditions. Due to their ease of interaction with biological systems, they have been widely employed in various therapeutic applications, including bone regeneration, stomach pain relief, and heartburn treatment. Green-synthesized MgO NPs exhibit a broad spectrum of biological activities, including antifungal, antibacterial, anticancer, and antioxidant effects. Their biodegradability, high cationic capacity, and redox properties contribute to their effectiveness in combating microbial infections, eradicating biofilms, and addressing antibiotic resistance (Thakur et al., 2022).

### 3.10 Safety and benefits of G-MNPs

Green-synthesized nanoparticles are typically functionalized with natural biomolecules, which enhance their biocompatibility and minimize cytotoxic effects. These biologically derived coatings facilitate a controlled and sustained release of metal ions, promoting the safe biodegradation and excretion of nanoparticles from the body while mitigating potential adverse effects (Thomas et al., 2023).

The green synthesis of green-synthesized metal nanoparticles (G-MNPs) presents substantial environmental and economic benefits. This approach is inherently cost-effective, scalable, and eco-friendly, as it minimizes the reliance on hazardous chemicals and reduces energy consumption. The inherent biodegradability of these nanoparticles further mitigates environmental impact, making them particularly suitable for applications requiring controlled degradation (Selvan et al., 2018). Compared to conventional synthesis methods, green synthesis offers a more economical alternative by eliminating the need for costly and toxic reagents, while the utilization of abundant biological resources further lowers production expenses (Gowda et al., 2022).

### 3.11 Mechanistic basis of G-MNPs in biomedical systems

The biological effectiveness of green-synthesized metal nanoparticles (G-MNPs), particularly in wound healing, antibacterial action, and anti-inflammatory therapy, is supported by their distinct physicochemical properties. Properties like surface charge, redox behavior, nanoscale size, and bifunctional surface ligands produced from microbial, or plant capping agents are important mechanisms (Guleria et al., 2022).

Because of their small size (usually between 10 and 100 nm), they can be efficiently taken up by cells by endocytosis, which allows for the targeted intracellular administration of reactive species or therapeutic substances. Both adhesion and internalization are impacted by the surface charge (zeta potential), which regulates electrostatic interactions with mammalian cell surfaces and microbial membranes. G-MNPs with a positive charge engage more strongly with negatively charged bacterial membranes, disrupting the membrane and killing the cell (Ahmed et al., 2016a).

Metal ions like Ag^+^ or Cu^2+^ in G-MNPs can catalyse the production of reactive oxygen species (ROS), such as superoxide and hydroxyl radicals, in terms of redox activity. Strong bactericidal actions are a result of the oxidative stress that harms the membranes, proteins, and DNA of microorganisms. By encouraging angiogenesis and fibroblast activation, ROS also affect wound healing at regulated doses (Kamaraj et al., 2024).

Further promoting tissue healing and immunomodulation are the anti-inflammatory and antioxidant qualities that phytochemical capping agents like flavonoids, terpenoids, and polyphenols provide. Through increased biocompatibility and less nonspecific protein adsorption, these surface ligands also lessen systemic toxicity (Karunakaran et al., 2023). G-MNPs are well suited for cutting-edge biomedical applications because of their combined physicochemical characteristics, which enable multifunctional therapeutic effects such as microbial clearance, inflammation suppression, oxidative balancing, and improved tissue regeneration (Soni et al., 2021).

### 3.12 Comparative ADME, in vivo fate, and toxicity profiles of G-MNPs

Understanding the absorption, distribution, metabolism, and excretion (ADME) behavior, as well as the *in vivo* degradation and toxicity of green-synthesized metal nanoparticles (G-MNPs), is vital for their safe biomedical application. Different metallic nanoparticles exhibit diverse biological interactions depending on their composition, size, surface chemistry, and capping biomolecules produced from green synthesis techniques. The ADME properties and biological impacts of widely utilized G-MNPs, such as iron oxide (FeONPs), zinc oxide (ZnONPs), silver (AgNPs), and gold (AuNPs), are contrasted in this section (Hosseingholian et al., 2023).

Safe biomedical use of green-synthesized metal nanoparticles (G-MNPs) requires an understanding of their toxicity, *in vivo* degradation, and absorption, distribution, metabolism, and excretion (ADME) behavior. The biological interactions of various metallic nanoparticles vary based on their size, content, surface chemistry, and capping biomolecules that come from green production methods. This section contrasts the ADME properties and biological impacts of widely utilized G-MNPs, such as iron oxide (FeONPs), zinc oxide (ZnONPs), silver (AgNPs), and gold (AuNPs) (Vijayaram et al., 2024).

Long circulation periods and delayed clearance are caused by the poor reactivity and chemical inertness of gold nanoparticles (AuNPs). According to biodistribution studies, the liver, spleen, and lymph nodes exhibit preferential accumulation. Hepatic routes and Kupffer cell phagocytic uptake are the main mechanisms in which they are cleared. Concerns regarding long-term biopersistence are raised by the fact that AuNPs are frequently kept in tissues longer than other metal NPs because of their stability. However, in green production, surface functionalization with biocompatible plant chemicals decreases the formation of protein corona and increases cellular absorption, increasing their usefulness in drug administration and imaging applications (Balasubramanian et al., 2010).

Because zinc oxide nanoparticles (ZnONPs) are partially soluble in physiological solutions, they exhibit special behavior. ZnONPs easily break down into Zn2+ ions, which are absorbed throughout the body and support metabolic processes. Ionic degradation decreases long-term buildup and increases their biodegradability. Usually, ZnONPs are eliminated through the stools and urine. Their biological effects include fibroblast proliferation and cytokine expression regulation; nevertheless, at larger concentrations, excessive ROS generation from Zn2+ may cause oxidative tissue damage. Green-synthesized ZnONPs with polyphenolic capping agents typically have stronger anti-inflammatory properties and less cytotoxicity (Smaoui et al., 2023).

Iron oxide nanoparticles (FeONPs) are known for their magnetic characteristics and therapeutic usage in imaging and hyperthermia. After administration, they are transported largely to the liver and spleen, where they are taken up by macrophages (Dadfar et al., 2019). Iron ions are released when FeONPs break down inside lysosomes and enter the body’s iron metabolic pathways, such as ferritin storage and hemoglobin formation. The risk of poisoning is greatly decreased by this natural metabolism. Good tolerance is shown in vivo investigations, particularly when the surface is functionalized with biocompatible coatings made from green synthesis, like flavonoids or tannins (Jacinto et al., 2025).

Green synthesis can reduce some toxicity by reducing chemical residue and improving biocompatibility, but careful control of dose, route of administration, and particle characteristics remains crucial. Further research involving systematic *in vivo* models, long-term biodistribution tracking, and mechanistic toxicology studies will be necessary for the safe clinical translation of G-MNPs. Overall, the *in vivo* fate and safety of G-MNPs are highly dependent on particle size, solubility, and surface properties conferred by the natural reducing and capping agents (Kyriakides et al., 2021).

## 4 Biomedical applications of G-MNPs

**FIGURE 3:**
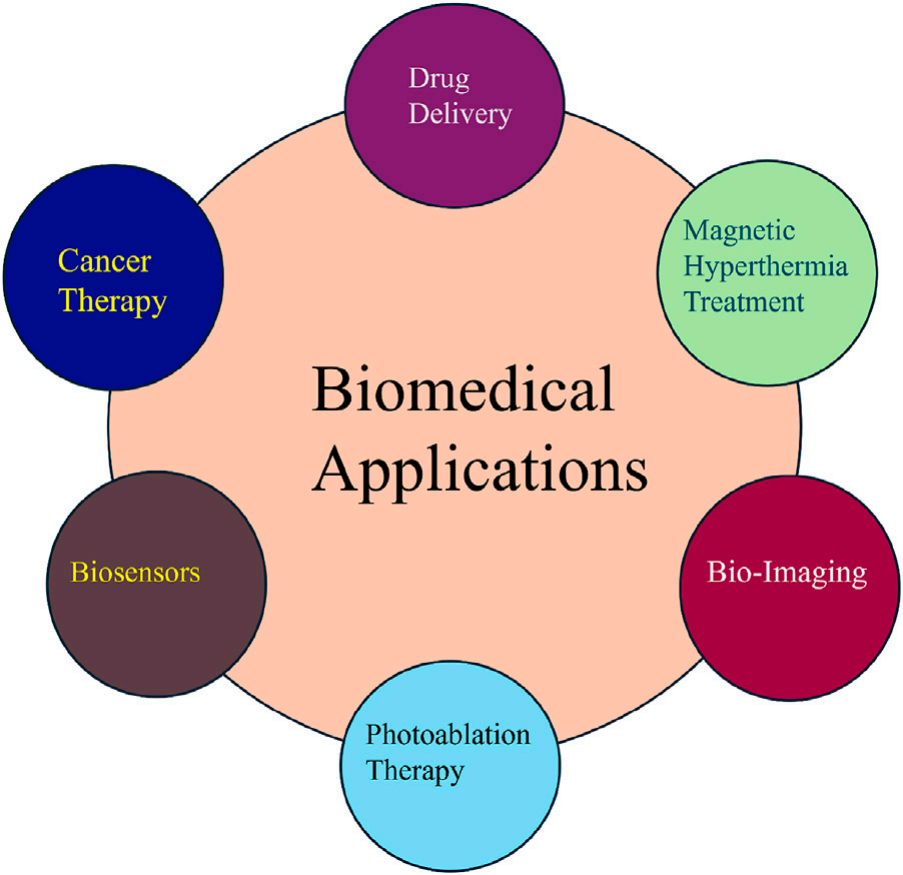
Biomedical application of G-MNPs.

In this section, we discuss the various biomedical applications of nanoparticles, focusing on their principles and specific uses, as outlined in Figure 3. Nanoparticles have significantly impacted biomedical engineering due to their distinct characteristics, including a high surface-to-volume ratio, unique optical, electronic, and magnetic properties, and enhanced surface energy. These attributes enable substantial modifications in pharmacokinetics, increased vascular circulation time, and improved bioavailability, especially for biomedical applications.

### 4.1 Drug delivery

Nanoparticles exhibit immense potential in drug delivery, particularly in enhancing drug efficacy and bioavailability while enabling reduced dosages compared to traditional bulk drugs. Targeted drug delivery, essential for minimizing damage to healthy tissues, particularly in cancer therapies, can be achieved by delivering drugs directly to tumor sites. Magnetic nanoparticles, particularly iron oxide, are commonly employed for this purpose, with other nanoparticles, such as silver (Ag), titanium dioxide (TiO2), iron-platinum (Fe–Pt), zinc oxide (ZnO), and gold (Au) nanoparticles, also demonstrating promise in drug delivery applications (Chatterjee et al., 2014). Nanoparticles’ high surface-to-volume ratio allows for extensive surface modifications that enhance drug release control, improve pharmacokinetics, and increase bioavailability. Surface modification is essential for targeted drug delivery and monitoring drug release, leveraging nanoparticles’ size-dependent optical, electronic, and magnetic properties. Magnetic nanoparticles are widely used in diagnostic imaging as MRI contrast agents, while optical properties enable the use of nanoparticles as alternatives to organic dyes for imaging (Kelly et al., 2003; Guskos et al., 2008). Nanoparticles also enhance target specificity and bio-membrane permeability, enabling them to be ideal drug delivery vehicles. Research continues to explore the use of nanoparticles for signal detection, transmission, and amplification, employing their magnetic, optical, and electronic properties (Arora et al., 2011). Core/shell nanoparticles, which provide additional advantages, are increasingly employed in biomedical applications. However, concerns regarding the toxicity of nanoparticles, including their penetration across bio-membranes and interference with basal metabolic processes, remain a significant challenge. Accumulation in the body due to the lack of efficient elimination mechanisms can result in severe conditions, including Alzheimer’s and Parkinson’s diseases, potentially leading to long-term health complications (Buzea et al., 2007).

### 4.2 Magnetic hyperthermia therapy

Magnetic hyperthermia (MH) represents a promising clinical approach for focal tumor treatment. This technique uses heat generated by magnetic nanoparticles when subjected to an alternating magnetic field (AMF) (Gilchrist et al., 1957). The advantages of MH, including high biosafety, deep tissue penetration, and selective tumor destruction, make it an attractive alternative to traditional cancer therapies (Ho et al., 2011). However, enhancing the efficiency of MH therapy remains a significant challenge, with particular focus on improving the thermal conversion efficiency of NPs. MH treatment involves heating tumors to temperatures above 42°C to induce cancer cell destruction, offering a targeted approach that spares surrounding healthy tissue. Iron oxide nanoparticles are commonly used for this application, but alternative NPs, such as bimetallic nanoparticles (Fe–Co, Cu–Ni) and other magnetic materials (Co–Fe2O4, Mn–Fe2O4), are also being explored (Liu et al., 2020).

For MH to be clinically viable, it is crucial to deliver adequate heat to the entire tumor while protecting healthy tissues. The therapeutic efficacy of MH is dependent on the magnetic susceptibility and thermal conversion efficiency of the NPs, with superparamagnetic iron oxide nanoparticles (SPIONs) being extensively studied for their biocompatibility (Hildebrandt et al., 2002; Vilas-Boas et al., 2020). Strategies to enhance thermal conversion efficiency include altering particle size (Mehdaoui et al., 2011), composition (Lee et al., 2011), shape (Lv et al., 2015), and surface characteristics (Liu et al., 2012). However, challenges remain due to the intrinsic limitations of NPs under AMF. Recent research suggests that localized induction heating at the nanoscale can modulate molecular properties, enhancing the effectiveness of MH therapy. MH is often used in combination with other cancer therapies, such as chemotherapy, radiotherapy, immunotherapy, and gene therapy, to improve treatment outcomes (Huang et al., 2010; Domenech et al., 2013).

### 4.3 Bioimaging

Medical imaging is essential for early disease detection and monitoring therapeutic responses. Existing imaging techniques include X-ray, CT, MRI, ultrasound, PET, SPECT, and fluorescence imaging. The integration of multiple imaging modalities is often used to enhance lesion detection. Conventional contrast agents, however, face limitations such as rapid metabolism, non-specific distribution, and potential toxicity (Torres Martin DE Rosales et al., 2011).

Nanoparticles have revolutionized medical imaging by providing unique passive, active, and physical targeting properties that enhance detection and imaging. Their small size enables enhanced permeability and retention (EPR) effects in tumors, increasing the concentration of contrast agents at tumor sites (Cuccurullo et al., 2018; Oh et al., 2013). The biodistribution and tumor penetration of nanoparticles are influenced by their size (Hoshyar et al., 2016; Scott and Quaggin, 2015; Longmire et al., 2008), with nanoparticles ranging from 10 to 60 nm being particularly effective for cellular uptake. Surface modifications with specific ligands further enhance nanoparticle targeting capabilities (Zhou and Dai, 2018; Huang et al., 2012). In addition to passive targeting, nanoparticles can be functionalized with targeting ligands, such as antibodies, aptamers, and peptides, to improve specificity for imaging applications (Kim et al., 2010; Wang et al., 2017; Alibakhshi et al., 2017; Jo and Ban, 2016; Alshaer et al., 2018). Techniques like gold nanoparticle-based CT imaging and superparamagnetic iron oxide nanoparticle-based MRI for lung cancer detection are examples of how nanoparticle surface modifications can be employed to enhance imaging contrast. External stimuli, such as light, magnetic fields, and ultrasound, can also be used to direct nanoparticle localization and control drug release. Nanoparticle-based imaging technologies are expected to play a significant role in non-invasive diagnostic and therapeutic applications (Inaba and Matsuura, 2019; Zhong et al., 2014; Yu et al., 2016; Wang et al., 2018; Yang et al., 2018).

### 4.4 Biosensors

Biosensors are analytical devices that detect biological samples and convert biological responses into electrical signals. These sensors must be highly specific, stable, and capable of analyzing biochemical reactions independently of external conditions. Nanoparticles enhance biosensor performance by increasing surface area for interaction, improving sensitivity, and enabling real-time monitoring of biological responses.

Biosensors are typically classified based on their transducing system, including calorimetric, potentiometric, optical, piezoelectric, and amperometric types. Nanoparticles play a critical role in enhancing biosensor sensitivity, especially in piezoelectric, amperometric, and optical sensors, by leveraging their inherent magnetic, electro-sensitive, and optical properties (Arora et al., 2011). For example, nanoparticles can improve the resolution and response time of Field-Effect Transistor (FET)-based biosensors. Research is focused on developing nanoparticle-based biosensors for specific applications, such as glucose detection and *in vivo* diagnostics, by functionalizing nanoparticles with enzymes, antibodies, or other sensing molecules. Core/shell nanoparticles are particularly useful in improving the catalytic activity and stability of biosensors (Chatterjee et al., 2014). Piezoelectric biosensors exploit the oscillatory properties of piezoelectric materials to detect changes in mass (Guilbault, 1983; Wang et al., 2006). These systems are highly sensitive and offer advantages such as solid-state construction, chemical inertness, and cost-effectiveness. Nanoparticles enhance frequency detection by increasing the mass on the crystal surface and leveraging the inherent piezoelectric properties of the nanoparticles (Zhang et al., 2010). Recent advancements include the use of Fe oxide/Au nanoparticles to detect volatile organic compounds and Fe3O4/Au nanocomposites for DNA mutation detection. These systems rely on localized surface plasmon resonance (LSPR) and piezoelectric signals to enhance sensitivity and specificity (Hayashi and Ruppin, 1985). Amperometric biosensors detect redox reactions by generating a current in response to electron transfer (Li et al., 2012). These sensors benefit from nanoparticle enhancements that improve catalytic activity and stability (Luo et al., 2007). Core/shell nanoparticles enhance charge transport efficiency and enable the development of portable, fast-response biosensors for *in-situ* diagnostics (Qiu et al., 2007; Zhang et al., 2007; Jimenez et al., 2008; Tan et al., 2009). Recent innovations include nanoparticle-based sensors for detecting metabolic substrates such as glucose and H_2_O_2_. These sensors are designed with core metal transducer nanoparticles and insulating shells to enhance performance (Gupta and Gupta, 2005; Chen et al., 2010).

Optical biosensors use light-sensitive nanoparticles, such as quantum dots and noble green-synthesized metal nanoparticles (G-MNPs) (e.g., gold and silver), to detect biological interactions (Gole et al., 2008). Nanoparticles offer superior surface functionalization capabilities and can be used in combination with magnetic cores for enhanced dispersibility and chemical stability (Pita et al., 2008). These sensors utilize phenomena like Surface Enhanced Raman Scattering (SERS) and Dipole Plasmon Resonance (DPR) to detect target molecules with high sensitivity (Endo et al., 2010). In conclusion, G-MNPs hold vast potential in biomedical applications, offering solutions to challenges in drug delivery, disease detection, and therapeutic interventions. Further research into their properties, modifications, and interactions within biological systems will continue to drive advancements in nanomedicine (Hamer et al., 2010).

### 4.5 Photoablation therapy

Photoablation therapy comprises two principal modalities: photodynamic therapy (PDT) and photothermal therapy (PTT). PDT leverages non-toxic, light-sensitive compounds known as photosensitizers, which exhibit cytotoxic properties upon activation by light of a specific wavelength. This approach is predominantly utilized for targeting diseased cells, including cancer cells (McNamara and Tofail, 2017). During PDT, photosensitizers such as TiO_2_ nanoparticles are exposed to light at a particular wavelength, leading to the generation of photo-induced electrons and holes. These charge carriers interact with water molecules or hydroxyl ions, producing highly reactive oxidative species, including reactive oxygen species (ROS) and singlet oxygen, thereby inducing cell death. In contrast, PTT employs near-infrared (NIR) light to irradiate tumor cells. The absorbed light energy is converted into heat, causing localized hyperthermia and resulting in cell death. TiO_2_ is an ideal candidate for PTT due to its biocompatibility, chemical stability, and intrinsic photocatalytic properties (Dougherty et al., 1998; Allison et al., 2006).

The photocatalytic mechanism of TiO_2_ involves three critical steps: excitation, diffusion, and surface transfer. Initially, TiO_2_ nanoparticles absorb photons from an external light source, imparting sufficient energy to overcome the material’s band gap and promoting electrons into the conduction band, leaving corresponding vacancies (holes) in the valence band. These electrons and holes subsequently diffuse to the surface of the photocatalyst. In the final stage, chemical reactions are triggered on the surface due to the presence of these charge carriers. The holes react with adsorbed water molecules to form hydroxyl radicals, while the electrons interact with oxygen to generate superoxide radicals. This cascade of photocatalytic reactions underpins the therapeutic effectiveness of TiO_2_ in both PDT and PTT (Yin et al., 2013).

### 4.6 Cancer therapy

Magnetic nanoparticles (MNPs) have emerged as a focal point of interest in the biomedical sciences due to their remarkable potential and diverse applications in nanotechnology. Their ability to form conjugates with ligands and drugs has led to a wide array of biomedical innovations, including magnetic separation, biotechnology, targeted drug delivery, analyte preconcentration, and diagnostic imaging. Nanomedicine, an interdisciplinary field integrating biomedicine, nanotechnology, and biomaterials, leverages MNPs as an innovative approach to address complex biomedical challenges (Quader and Kataoka, 2017). MNPs are particularly advantageous for cancer treatment due to their precise and tunable properties, such as size, shape, charge, and surface modifications. These nanoparticles exhibit enhanced cellular uptake compared to non-metallic nanoparticles of equivalent size, providing a distinct benefit for targeted cancer therapy (Evans et al., 2018). The use of MNPs in biomedicine dates back to 1857, when Michael Faraday first described the synthesis of silver nanoparticles (AgNPs) in aqueous solutions, which led to the formation of a ruby-colored solution upon reaction with gold salt (Faraday, 1996).

The unique physicochemical properties of MNPs, including a high surface area-to-volume ratio, enhanced surface energy (El-Sayed, 2001), surface plasmon resonances (SPR), abundant dangling bonds, electron storage capacity, and the presence of sharp edges and corners, render them highly suitable for biomedical applications. MNPs can be synthesized using a range of techniques, including physical, chemical, and biological methods (Abdal Dayem et al., 2018). Pure MNPs include materials such as silver, gold, and copper, while metal oxide nanoparticles, such as titanium dioxide, silica, zinc oxide, and iron oxide, are also employed in various pharmaceutical and biomedical applications. However, the high surface energy of MNPs can lead to metal-metal aggregation, posing challenges in maintaining stable colloidal solutions. Ongoing efforts are focused on developing strategies to enhance the stability and functionality of MNPs, thereby maximizing their biomedical utility (Khursheed et al., 2022).

**FIGURE 4:**
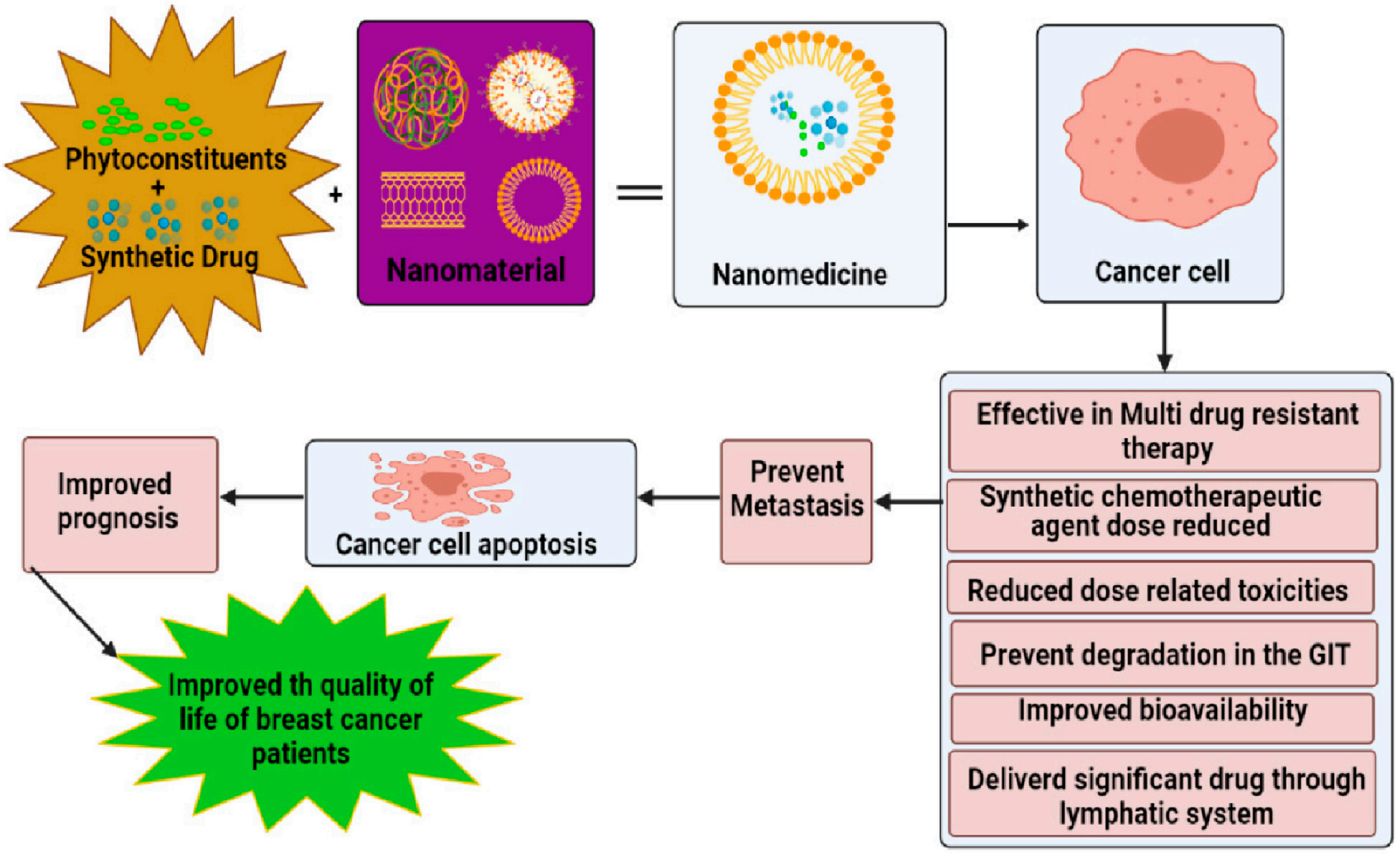
Schematic representation of nanomedicine combinatorial approaches on cancer cells and its effectiveness (Tagde et al., 2022).

MNPs can be utilized for both passive and active targeting in drug delivery systems. In passive targeting, the rapid growth of solid tumors often results in poor lymphatic drainage and aberrant vasculature, enabling MNPs to accumulate at tumor sites through the fenestrations in the circulatory system. This phenomenon, known as the enhanced permeability and retention (EPR) effect, facilitates the preferential accumulation of nanoparticles within tumor tissues (Gil and Parak, 2008). Surface functionalization of nanoparticles with hydrophilic moieties, such as polyethylene glycol (PEG), enhances their solubility, reduces macrophage uptake, prevents premature elimination from circulation, and offers protection against enzymatic degradation during *in vivo* studies. For active targeting, nanoparticles can be functionalized with specific targeting ligands, such as antibodies, which bind to tumor-specific receptors or surface proteins. This approach facilitates selective targeting of cancer cells, thereby enhancing the therapeutic efficacy of the encapsulated drugs while minimizing damage to healthy tissues. Numerous studies have demonstrated the promising therapeutic outcomes of drug-loaded MNPs in cancer treatment, underscoring their potential to improve the precision and effectiveness of cancer therapies (Conde et al., 2012). Figure 4 provides a schematic representation of novel MNP-based drug delivery strategies for cancer treatment (Tagde et al., 2022).

## 5 Biological benefits and mechanistic understanding of green-synthesized metal nanoparticles in wound healing

Green-synthesized metal nanoparticles (G-MNPs) have attracted a lot of attention because of their functional biocompatibility, eco-friendly production, and inherent bioactivity. G-MNPs’ ability to heal wounds is aided by the retention of bioactive components from the biological source (plants, microorganisms, and algae), such as polyphenols, flavonoids, terpenoids, and alkaloids, in contrast to chemically or physically manufactured nanoparticles.

### 5.1 Reduced cytotoxicity and improved biocompatibility

G-MNPs’ cytotoxicity to mammalian cells is much decreased when they have natural capping agents on them. According to studies, when compared to their chemically synthesized counterparts, silver and gold nanoparticles made with extracts from Azadirachta indica, Aloe vera, or Camellia sinensis show less oxidative stress and greater fibroblast compatibility in skin models (Ahmed et al., 2016a, Mostafavi and Shabani, 2024).

### 5.2 Antioxidant and anti-inflammatory properties

At the wound site, oxidative stress and inflammatory cytokines are actively modulated by bioactive phytochemicals incorporated in G-MNPs. For instance, green-synthesized ZnO and AgNPs made with extracts from Curcuma longa or Ocimum sanctum show inhibition of ROS formation, IL-1β, and TNF-α, which speeds up the shift from inflammatory to proliferative wound healing phases (Hamed et al., 2023).

### 5.3 The effectiveness of antibiotics

To reduce infection-related problems throughout the healing process, green-synthesised AgNPs and CuNPs have shown broad-spectrum antibacterial action against *Staphylococcus aureus*, *E. coli*, and *Pseudomonas aeruginosa*. Interestingly, both metal ion release and phytochemical components are responsible for the synergistic antibacterial activity (Pourmadadi et al., 2024).

### 5.4 Tissue regeneration and angiogenesis

Certain G-MNPs encourage angiogenesis, which is essential to produce granulation tissue and the delivery of nutrients. For example, in excisional wound models, gold nanoparticles made with leaf extract from Salvia officinalis markedly enhanced capillary development and VEGF expression. G-MNPs also promote collagen deposition, fibroblast migration, and re-epithelialization (Cucci et al., 2021).

## 6 Translational relevance of green-synthesized metal nanoparticles (G-MNPs) in wound care

Developed utilizing plant extracts, microbial agents, or natural biomolecules, green-synthesized metal nanoparticles (G-MNPs) have shown great promise as wound healing nanotherapeutics because of their environmentally friendly synthesis, multifunctional therapeutic benefits, and positive safety profiles. There is increasing evidence from *in vitro*, *in vivo*, and early preclinical research that they can address important therapeutic difficulties such tissue regeneration, inflammation, and infection control, indicating their translational value in wound care.

### 6.1 Biocompatibility and safety advantage

Biological entities like plant extracts, fungus, or bacteria that include naturally occurring reducing and stabilizing chemicals like flavonoids, alkaloids, terpenoids, polyphenols, and proteins are used to create green-synthesized metal nanoparticles (G-MNPs). In addition to aiding in the production of nanoparticles, these biomolecules also stabilize and cap their surfaces, greatly increasing their biocompatibility and lowering the possibility of cytotoxicity. Better integration with host tissues, less inflammatory response, and enhanced cellular connections are all facilitated by this bio-functional surface layer (Aigbe and Osibote, 2024). On the other hand, chemically produced nanoparticles frequently contain residual hazardous chemicals (such as hydrazine and sodium borohydride) that might cause immunogenic reactions, oxidative stress, or damage to cell viability. Because of its safer profile, G-MNPs are especially well-suited for applications involving cutaneous wounds, where it is impossible to avoid direct contact with delicate tissue. When compared to chemically synthesized silver nanoparticles, for instance, silver nanoparticles made with Azadirachta indica (neem) leaf extract demonstrated significantly higher fibroblast proliferation, antimicrobial activity, and wound closure rate in vivo models, demonstrating the dual advantages of metallic ion activity and bioactive phytochemical synergy (Singh et al., 2023).

### 6.2 Enhanced therapeutic functions

In the wound healing cascade, green-synthesized metal nanoparticles (G-MNPs) provide a multifunctional therapeutic profile that is very advantageous. These nanoparticles have a variety of bioactivities, such as angiogenic, antibacterial, anti-inflammatory, and antioxidant qualities, all of which are essential for encouraging tissue regeneration and repair. The bioactive substances found in the biological materials employed during synthesis frequently enhance the therapeutic potential of G-MNPs. In addition to the intrinsic qualities of the metal core, phytochemicals like polyphenols, flavonoids, terpenes, and alkaloids can stay adsorbed on the surface of the nanoparticle and have synergistic effects (Malik et al., 2025). By interfering with microbial membranes and biofilms, G-MNPs’ antimicrobial effect aids in preventing infection, which is a significant obstacle to wound healing. By lowering levels of pro-inflammatory cytokines like TNF-α and IL-6, their anti-inflammatory properties help to modulate the early inflammatory phase of healing and avoid chronic inflammation. As free radical scavengers, G-MNPs also promote cellular migration and proliferation while reducing oxidative stress in the wound microenvironment. Some metal nanoparticles, especially zinc oxide and gold, have angiogenic qualities that aid in tissue remodeling by encouraging neovascularization, which is necessary for the transport of nutrients and oxygen (Abuzeid et al., 2023). For example, bioactive curcuminoids, which have strong anti-inflammatory and antioxidant qualities, were preserved on the surface of gold nanoparticles made from Curcuma longa (turmeric). These nanoparticles greatly reduced pro-inflammatory cytokines like TNF-α and IL-6 in a diabetic wound model while encouraging tissue remodeling and epithelial regeneration, which sped up the healing process (Thiruvengadam et al., 2025).

### 6.3 Innovative formulations and delivery systems

Green-synthesized metal nanoparticles (G-MNPs) have been progressively included into a variety of cutting-edge wound care delivery methods in an effort to improve clinical translation and therapeutic efficacy. These consist of topical films, hydrogels, electrospun nanofibers, nanogels, and bio-composite dressings. In addition to acting as transporters, these formulations also act as useful scaffolding that actively aid in the healing of wounds. These delivery systems’ capacity to transport metal ions and nanoparticles in a controlled and maintained manner, guaranteeing a longer therapeutic effect at the wound site, is one of its main advantages. Furthermore, by keeping the environment moist, these systems promote cellular growth and re-epithelialization while thwarting microbial invasion and desiccation (Faghani and Azarniya, 2024). These materials frequently provide the wound bed with mechanical and structural support in addition to medication release. For example, fibroblast adhesion, migration, and the development of new tissue can be encouraged by electrospun nanofibers that imitate the extracellular matrix (ECM). For chronic and non-healing wounds, hydrogels—especially those derived from biopolymers like chitosan or alginate—offer exceptional biocompatibility and can be customized to react to the pH, temperature, or enzymatic activity of the wound (Liang et al., 2023). The addition of zinc oxide nanoparticles made with Aloe vera extract to a hydrogel matrix based on chitosan is a noteworthy illustration of this strategy. When tested on rat burn wound models, this composite dressing showed markedly faster wound contraction, increased angiogenesis, and decreased inflammation. Aloe vera’s inherent healing qualities, the bioactivity of ZnO nanoparticles, and the chitosan hydrogel’s moisture-retentive and biocompatible qualities were all credited with these therapeutic benefits. When included into cutting-edge wound dressings that satisfy both therapeutic and clinical usability requirements, such novel systems highlight the translational potential of G-MNPs(Alvandi et al., 2024).

### 6.4 Sustainability and cost-effectiveness

A significant benefit of green-synthesized metal nanoparticles (G-MNPs) is their intrinsic economic viability and environmental sustainability, which makes them particularly appealing for clinical application in wound care, especially in environments with limited resources or low and middle incomes. As reducing and stabilizing agents, green synthesis uses naturally occurring materials like plant extracts, microbes, or agricultural waste, in contrast to traditional chemical or physical synthesis procedures that frequently call for significant energy inputs, hazardous solvents, and costly reagents. These biodegradable, renewable, and plentiful biological inputs greatly lower the environmental impact and production costs associated with the creation of nanoparticles (Aigbe and Osibote, 2024). Additionally, the green synthesis process can be scaled up in relatively mild conditions (ambient temperature and pressure), reducing the need for energy and infrastructure. For the industrial development of wound care solutions based on nanoparticles, which must be manufactured in large quantities without sacrificing efficacy or safety, scalability is essential. Furthermore, the low production of dangerous byproducts supports international objectives for green and sustainable nanotechnology, guaranteeing legal compliance and public health safety (Aigbe and Osibote, 2024). For example, Radulescu et al. (2023) found that the synthesis of plant-based nanoparticles supports environmentally aware, financially feasible clinical development by lowering the burden of disposing of hazardous waste and reducing the requirement for expensive synthetic chemicals. In this regard, G-MNPs offer a viable approach to creating accessible and reasonably priced wound care technologies with few legal and environmental restrictions (Radulescu et al., 2023).

### 6.5 Current limitations and path forward

Green-synthesized metal nanoparticles (G-MNPs) have shown promising therapeutic results and environmental benefits; nonetheless, several significant obstacles still stand in the way of their broad clinical application. The heterogeneity of synthesis procedures, especially when employing plant or microbial extracts, is one of the main obstacles. Batch-to-batch variations in nanoparticle size, shape, surface chemistry, and biological activity might result from the substantial variation in composition of these biological sources based on species, season, place of origin, and extraction conditions. These discrepancies make it challenging to meet regulatory requirements and produce repeatable therapeutic results (Kurul et al., 2025). A significant obstacle is the absence of thorough long-term safety data. The systemic toxicity, immunogenicity, biodegradation, and clearance profiles of G-MNPs over extended periods of time are still poorly understood, despite the fact that numerous studies have shown short-term biocompatibility and efficacy *in vitro* and in small animal models. Standardized *in vivo* testing frameworks and well planned preclinical research are required to fill in these toxicological data gaps (Kurul et al., 2025). Furthermore, the approval of nanomaterials originating from natural sources is surrounded by regulatory ambiguity. Uncertainty over classification, documentation requirements, and safety validation processes arises from regulatory authorities’ frequent absence of particular rules suited to green nanomaterials. This is a problem for both market authorization and large-scale manufacture (Singh et al., 2024). However, encouraging progress is being made to get over these obstacles. More control over the quality and consistency of nanoparticles is becoming possible thanks to developments in standardized synthesis processes, green chemistry validation frameworks, and high-resolution nanoparticle characterization techniques (such as TEM, DLS, FTIR, and XPS). Additionally, the incorporation of G-MNPs into translational pipelines is being expedited by interdisciplinary partnerships among materials scientists, toxicologists, and regulatory specialists. It is anticipated that these initiatives will soon open the door for safe, efficient, and economically feasible G-MNP-based wound care solutions with sustained research funding and policy development (Mulla, 2024).

## 7 Future perspectives

An inventive and sustainable development in nanomedicine is the incorporation of green-synthesized metal nanoparticles (G-MNPs) into wound healing applications. Because of their natural biocompatibility, antibacterial activity, and tissue-regenerative properties, these nanoparticles which come from biological sources like plant extracts, fungus, and bacterial metabolites are excellent choices for next-generation wound care. Table 2 lists the therapeutic advantages of G-MNPs, including less cytotoxicity, improved healing kinetics, and environmentally friendly manufacturing (Iravani, 2011).

**TABLE 2:** Specific biological sources and their functional advantages in green-synthesized nanoparticles for wound healing.

Biological source	Nanoparticle synthesized	Bioactive components	Functional advantage in wound healing
*Azadirachta indica* (Neem) leaf extract	Silver nanoparticles (AgNPs)	Flavonoids, terpenoids, nimbin	Enhanced antimicrobial activity, fibroblast proliferation, and faster wound closure in rats (Dutt et al., 2023)
*Aloe vera* leaf extract	Zinc oxide nanoparticles (ZnONPs)	Anthraquinones, acemannan	Promoted collagen synthesis, re-epithelialization, and anti-inflammatory effect (Lou and Chen, 2023)
*Curcuma longa* (Turmeric) rhizome extract	Gold nanoparticles (AuNPs)	Curcumin, polyphenols	Potent anti-inflammatory and antioxidant properties, reduced TNF-α expression (Wang et al., 2022)
*Calotropis gigantea* latex	AgNPs	Cardiac glycosides, tannins	Accelerated wound contraction and epithelial regeneration in excision wound models (Banerjee and Ravishankar Rai, 2018)
*Terminalia arjuna* bark extract	AuNPs	Ellagic acid, flavonoids	Enhanced angiogenesis and granulation tissue formation (Majoumouo et al., 2020)
*Trichoderma harzianum* (fungus)	AgNPs	Secondary metabolites (e.g., peptaibols)	Inhibited pathogenic biofilms and supported fibroblast migration (Vanlalveni et al., 2024)
*Lactobacillus plantarum* (probiotic bacteria)	AgNPs	Lactic acid, bacteriocins	Promoted antimicrobial action and skin barrier recovery (Chen et al., 2024)

The mechanisms of wound healing and bactericidal activity of various green-synthesized metal nanoparticles (G-MNPs) are summarized in Table 3. The advantages and disadvantages associated with G-MNPs are presented in Table 4, while the mechanistic benefits of green synthesis in wound healing applications are detailed in Table 5. A summary of representative translational outcomes of G-MNPs is provided in Table 6, and the beneficial effects of green-synthesized metal nanoparticles in wound healing are highlighted in Table 7.

**TABLE 3:** Mechanism of wound healing and bactericidal activities of different G-MNPs.

Plant materials	Nanoparticles	Wound healing mechanism	Bactericidal mechanism
*Citrus lemon*	Ag-NP Ag-NP + Chitosan + propolis extract	Fibroblast proliferation, collagen synthesis, and angiogenesis play critical roles in the wound healing process (Abbasi et al., 2021). The reduction in wound size can be attributed to the antibacterial and anti-inflammatory properties of bioactive components, which mitigate microbial contamination, facilitate tissue regeneration, and promote the restoration of structural integrity. By preventing infection and modulating inflammatory responses, these components contribute to an accelerated and more efficient healing process (Al-Shmgani et al., 2017)	- Disruption of the bacterial cell membrane induces membrane permeabilization, leading to the leakage of intracellular contents, loss of cellular integrity, and ultimately, bacterial cell death (Al-saggaf, 2021)
*Ilex paraguariensis*	Electrospun polyacrylic acid and polyallylamine hydrochloride loaded ZnONP	-	Damage to the cell membrane, followed by the internalization of nanoparticles, metal ions, and reactive oxygen species (ROS), disrupts cellular homeostasis and adversely impacts metabolic processes, ultimately compromising cell viability (Sangeetha et al., 2011; Nava et al., 2017)
*Aloe barbadensis*	ZnONP + Silica gel	Enhanced platelet activation, apoptosis, tissue necrosis, angiogenesis, re-epithelialization, and stem cell activation are key processes that facilitate wound repair and tissue regeneration (Batool et al., 2021)	Accumulation of ZnO NPs and the production of ROS (Batool et al., 2021)
*Azadirachta indica*	AgNP + PF127 hydrogel	Remodeling and re-epithelialization (Liu et al., 2010)	Damage to the cell membrane results in cytoplasmic contraction and the subsequent efflux of intracellular contents (Chinnasamy et al., 2021)
*Lawsonia inermis L*	AgNP + Talc + Chitosan	Modulation of gene expression leads to the induction of the anti-inflammatory M2 macrophage phenotype, characterized by upregulation of markers such as CD206, bFGF, IL-10, and collagen type I (Collagen1A), which collectively promote fibroblast migration and tissue repair (Daghian et al., 2021)	Chitosan interacts with cell membranes, enhancing their permeability and leading to bacterial cell death through membrane disruption (Tang et al., 2010)
*Parkia biglandulosa*	AgNP	Supported cell proliferation (John et al., 2021)	Destabilization of the bacterial outer membrane results in rupture of the plasma membrane and induces alterations in the physical and chemical properties of both the cell wall and membrane. The interaction of silver nanoparticles (AgNPs) with sulfur-containing membrane proteins or phosphorus-containing DNA, along with the release of silver ions, contributes to the destruction of bacterial cells (John et al., 2021)
*Boletus edulis (Mushroom)*	AgNP	Migration of fibroblasts (Kaplan et al., 2021)	Release of reactive oxygen species (ROS) disrupts the electron transport chain and compromises cellular integrity by interacting with phosphorus and sulfhydryl groups in the cell wall (Sepehri et al., 2021)
*Coriolus versicolor (Mushroom*	AgNP TiO2 + heparin-polyvinylalcohol (H-PVA)	Migration of fibroblasts (Kaplan et al., 2021) Titanium dioxide (TiO2) interacts with bacterial cell walls, leading to membrane disruption, leakage of cellular contents, and subsequent bacterial cell death (Sonamuthu et al., 2020)	The release of reactive oxygen species (ROS) interferes with the electron transport chain, compromising cellular integrity by reacting with phosphorus and sulfhydryl groups within the cell wall (Sepehri et al., 2021) Fibroblast migration, epithelial cell proliferation, and the restoration of blood flow are facilitated through the formation of new blood vessels, promoting tissue repair and regeneration (Pangli et al., 2021)
*Echinophora platyloba* *DC*	AgNP + Chloroxine	Enhanced re-epithelialization, reduced wound inflammation, and modulation of fibrogenic cytokine expression contribute to the promotion of wound healing (Shahabadi et al., 2021)	Penetrating the bacterial cell, the agent interacts with and damages sulfur- and phosphorus-containing biomolecules, such as DNA (Shahabadi et al., 2021)
*Scutellaria barbata*	AgNP AgNP + poly (carboxybetaine- odopamine methacrylamide) (PCBDA) copolymer	Induction of fibroblast cell proliferation, differentiation and migration (Veeraraghavan et al., 2021) Deposition of collagen and re-epithelization (Xiang et al., 2021)	Silver cations disrupt bacterial cells by binding to thiol groups in bacterial proteins, leading to structural and functional impairment, ultimately resulting in cell death (Radzig et al., 2013) Contact-killing damages the cell membrane and kills the bacteria (Xiang et al., 2021)
*Prosopis cineraria*	ZnPC	Collagen synthesis, re-epithelialization, and neovascularization are promoted alongside an increase in fibroblast cell proliferation. Furthermore, wound healing is enhanced by the synergistic effects of the anti-inflammatory phenolic compounds present in Prosopis cineraria and zinc oxide (ZnO) (Yadav et al., 2021)	-
*Prosopis cineraria*	FePC	Collagen formation, re-epithelialization, and keratinization play crucial roles in the wound healing process. Additionally, wound repair is facilitated by the synergistic anti-inflammatory effects of phenolic compounds derived from Prosopis cineraria and iron oxide (Fe_3_O_4_) (Yadav et al., 2021)	-
*Phormidium* sp. *(cyanobacterium)*	AgNP AgNP + iturin + chitosan	Re-epithelialization and cytokine modulation play essential roles in wound healing. The repair process is further enhanced by the upregulation of enzymatic antioxidants and the suppression of pro-inflammatory cytokines, promoting a balanced healing response (Younis et al., 2021) Collagenation and re-epithelialization (Zhou et al., 2021)	Silver ions interact with bacterial DNA, inhibiting essential enzymatic functions, while silver nanoparticles (AgNPs) induce structural damage to the cell wall and cytoplasmic membrane, ultimately compromising bacterial viability (Naraginti et al., 2016) -

**TABLE 4:** Advantages and disadvantages of G-MNPs.

Nanoparticle type	Source	Advantages	Disadvantages	Safety insights
AgNPs (Silver)	*Azadirachta indica*	Strong antimicrobial, anti-inflammatory	ROS overproduction at high dose	Hemocompatible at ≤50 μg/mL (Ahmed et al., 2016a)
AuNPs (Gold)	*Ocimum sanctum*	Biocompatible, easy to functionalize	High cost of gold salts	No cytotoxicity at 10–100 μg/mL (Ankamwar et al., 2005)
ZnONPs (Zinc Oxide)	*Aloe vera*	Promotes wound healing, enhances fibroblast migration	Instability in aqueous media	Mild oxidative stress observed (Wu et al., 2024)
CuONPs (Copper Oxide)	*Tridax procumbens*	Angiogenesis stimulation	Risk of copper ion leaching	Requires dose-controlled use (Barapatre et al., 2016)

**TABLE 5:** Mechanistic advantages of green-synthesized MNPs in wound healing.

Type of G-MNP	Biological source	Key mechanism	Observed outcome
AgNPs	*Azadirachta indica*	Antimicrobial, fibroblast proliferation	Faster wound closure (Singh et al., 2018)
ZnONPs	*Ocimum sanctum*	Anti-inflammatory, ROS reduction	Enhanced epithelialization (Hamed et al., 2023)
AuNPs	*Salvia officinalis*	Angiogenesis via VEGF upregulation	Improved granulation (Cucci et al., 2021)
CuNPs	*Green algae* sp	Broad-spectrum antibacterial	Reduced infection at wound site (Pourmadadi et al., 2024)

**TABLE 6:** Representative translational examples.

G-MNP type	Biological origin	Formulation	Wound model	Translational outcome
AgNPs	*Azadirachta indica* (Neem)	Hydrogel dressing	Excision wound (rat)	Enhanced wound closure, low inflammation (Dutt et al., 2023)
AuNPs	*Curcuma longa* (Turmeric)	Topical cream	Diabetic wound (mouse)	Reduced cytokine levels, better tissue regeneration (Abbas, 2021)
ZnONPs	*Aloe vera*	ZnO–chitosan hydrogel	Burn wound (rat)	Promoted fibroblast migration, angiogenesis (Wafi and Khan, 2024)

**TABLE 7:** Beneficial effects of green-synthesized metal nanoparticles in wound healing.

Beneficial effect	Mechanism & impact
Antimicrobial Properties	G-MNPs disrupt microbial cell walls, inhibit biofilm formation, and prevent infections (Shaikh et al., 2019)
Anti-Inflammatory Effects	Reduce reactive oxygen species (ROS), modulate cytokines, and suppress chronic inflammation (Al-Khattaf, 2021)
Enhanced Cell Proliferation & Tissue Regeneration	Stimulate fibroblast proliferation, angiogenesis, and collagen synthesis (Ahmed et al., 2016b)
Controlled Drug Release	Functionalized G-MNPs enable targeted and sustained delivery of therapeutic agents (Iravani et al., 2014)
Reduced Cytotoxicity & Environmental Impact	Use of biocompatible natural reducing agents lowers toxicity and environmental burden (Mittal et al., 2013)
Cost-Effectiveness and Sustainability	Plant/microbial-based synthesis methods offer low-cost, scalable, and green production (Iravani, 2011)
Smart Wound Care Applications	Responsive nanomaterials support real-time monitoring and personalized treatment (Kaushik et al., 2023)

Despite these encouraging aspects, clinical translation and widespread commercialization are hampered by several significant issues. Among these, metabolic clearance, *in vivo* biodistribution, long-term cytotoxicity, and batch-to-batch repeatability are crucial. Different species, seasons, and environmental factors can affect the phytochemical makeup of plant and microbial extracts, leading to variations in the size, shape, and surface chemistry of the nanoparticles. Thus, it is still vital to standardize biological supplies and optimize reaction parameters. To guarantee controlled and predictable synthesis results, future research must give top priority to the development of high-throughput screening, extract fingerprinting, and enzyme-specific reduction investigations (Bhainsa and D’souza, 2006).

Standardized *in vitro* and *in vivo* models and longitudinal toxicity evaluations are crucial for assessing the immunogenicity and safety profile of G-MNPs (Mittal et al., 2013). In this sense, real-time nanoparticle tracking and wound monitoring can be facilitated by sophisticated imaging techniques (such as fluorescence, MRI, and photoacoustic). Furthermore, regulatory channels present formidable obstacles. Before approving a treatment, regulatory bodies such as the FDA, EMA, and ISO want comprehensive nanoparticle characterization, stability testing, and extensive clinical trials to prove efficacy and safety. Future research should concentrate on developing consensus frameworks for evaluating green nanoparticles using databases on nanotoxicology and international regulatory requirements (Ahovan et al., 2022).

From a technological and manufacturing standpoint, the scalability of green synthesis remains constrained due to manual processing, extract variability, and a lack of continuous systems. Transitioning to automated and bioreactor-based production systems can greatly improve output, uniformity, and commercial viability. Developments in enzyme-catalysed reduction and microbial-assisted synthesis provide new opportunities for fine-grained control over the shape and functionality of nanoparticles (Bamidele et al., 2025).

A promising area for the future is the creation of intelligent wound dressings that use stimuli-responsive G-MNPs. When functionalized with growth factors, antimicrobial peptides, or environmental sensors, these smart nanomaterials can monitor infection, deliver targeted medicines, and dynamically adjust to wound conditions. G-MNPs and biopolymers like collagen, chitosan, or cellulose can work together in hybrid wound dressings to improve tissue regeneration, antibacterial activity, and anti-inflammatory benefits. Hydrogels, electrospun nanofibers, and three-dimensional scaffolds are examples of nanocomposite platforms that have demonstrated promise in enhancing oxygen exchange, moisture retention, and prolonged medication release all crucial factors for successful chronic wound care, particularly in diabetic burns and ulcers (Moradifar et al., 2025; Nasra et al., 2024).

Furthermore, there is revolutionary potential in incorporating machine learning (ML) and artificial intelligence (AI) into wound therapy and nanoparticle creation. AI-driven models can assist in real-time therapeutic decision-making, anticipate ideal synthesis conditions, and customize nanoparticle properties for certain wound types. Personalized medicine approaches to wound care may be made possible by AI-enabled biosensors integrated in wound dressings that allow for continuous monitoring of wound pH, infection biomarkers, and healing rate (Shankhwar et al., 2025).

Despite the obvious environmental benefits of green synthesis, life cycle analyses and eco-toxicological evaluations are essential to guaranteeing the safety and sustainability of large-scale production. To reduce possible environmental hazards, scientific innovation should be accompanied by research into waste reduction, biodegradable capping agents, and ethical biomaterial sourcing (Osman et al., 2024).

In conclusion, G-MNPs offer a strong foundation for creating multipurpose, environmentally responsible, and clinically successful wound healing therapies. However, interdisciplinary cooperation between materials science, microbiology, pharmacology, clinical medicine, and regulatory science is necessary to realize their full potential. To move G-MNP-based wound care solutions from the lab to international clinical practice, it will be crucial to prioritize standardization, safety validation, AI-driven design, and sustainable manufacturing.

## 8 Conclusion

Green-synthesized metal nanoparticles (G-MNPs) exhibit remarkable versatility across a wide array of applications, including energy harvesting, microelectronics, agriculture, food science, and medicine. Traditionally, their synthesis has involved physical, chemical, and biological pathways. However, green synthesis techniques have emerged as a particularly attractive alternative due to their economic viability, non-toxicity, and environmental benefits. This comprehensive analysis compiles critical data on the synthesis, characterization, and applications of G-MNPs. It explores into their metal-toxicity, antioxidant, anticancer, antifungal, antimalarial, and photocatalytic properties. The findings significantly support the use of green synthesis methods to enhance the potential of MNPs in biomedicine and environmental applications.

This review paper underscores the feasibility of nanoscale metal synthesis using various plant sources, discussing the green synthesis of Au, Ag, Fe, Cu, and Pd MNPs. Despite significant progress, several challenges remain, such as limited yield, size heterogeneity, complex extraction procedures, and fluctuations in raw material supply due to seasonal and regional factors. Addressing these challenges requires further research on improving particle yield, using cost-effective starting materials, and incorporating energy-efficient technologies. MNPs are widely used for their antimicrobial properties against bacteria, fungi, and certain viruses, attributed primarily to the metal component, although bio-MNPs also contain vital biomolecules. These antimicrobial properties are utilized in various industries, including food packaging, skincare products, disease treatment, and drug delivery. However, it is important to note that the overuse and extensive deployment of MNPs could lead to toxicity due to the accumulation of metals and ions. Despite this potential, no lethal effects on humans at the currently used concentrations have been reported. In conclusion, green synthesis represents a largely positive and significant advancement across all scientific fields. The use of environmentally friendly resources and biodegradable materials in the synthesis of MNPs is poised to usher in an eco-friendly era with reduced industrial and environmental pollution.

Singh et al. (2018) used a murine excision model to compare citrate-stabilized AgNPs with AgNPs made using Azadirachta indica extract. The G-AgNP group showed reduced inflammatory infiltration, increased collagen alignment (Masson’s trichrome), and markedly improved wound contraction. Chemically produced AgNPs, on the other hand, resulted in minor cutaneous irritation and delayed granulation.

In a similar vein, Hamed et al. (2023) showed that ZnONPs made with Ocimum sanctum were more effective than ZnONPs made by chemical precipitation at modulating pro-inflammatory cytokines and oxidative stress indicators (GSH, SOD).

These illustrations show that green synthesis is not just an environmentally benign method; it also profoundly modifies the biological interface and surface functionality of nanoparticles, improving their ability to heal wounds.

## Glossary

G-MNPs: Green-synthesized metal nanoparticles
UV-Vis: Ultraviolet-visible
SPR: Surface plasmon resonance
FTIR: Fourier Transform Infrared
ATR: Attenuated total reflectance
CCDs: Charge-coupled devices
O: Oxygen
H: Hydrogen
N: Nitrogen
IR: Infrared
SERS: Surface-enhanced Raman spectroscopy
CARS: Coherent anti-Stokes Raman spectroscopy
TERS: Tip-enhanced Raman spectroscopy
ICP-MS: Inductively Coupled Plasma Mass Spectrometry
TOF: time-of-flight
NMR: Nuclear Magnetic Resonance
RF: Radiofrequency
keV: Kilo electron Volt
SAED: Selected Area Electron Diffraction
EDS: Energy-Dispersive X-ray Spectroscopy
SEM: Scanning Electron Microscopy
AFM: Atomic Force Microscopy
TGA: Thermogravimetric Analysis
XRF: X-ray fluorescence
DSC: ifferential Scanning Calorimetry
TEM: Transmission Electron Microscopy
XRD: X-ray Diffraction
CVD: Chemical vapor deposition
NADH: Nicotinamide adenine dinucleotide (NAD) + hydrogen (H)
Hg: Mercury
3D: Three Dimensional
4D: Four Dimensional
MRSA: methicillin-resistant *Staphylococcus aureus*
TNF-α: Tumor necrosis factor alpha
IL: Interleukin
VEGF: Vascular endothelial growth factor
ECM: Extracellular matrix
COL1: Collagen Type 1
COL3: Collagen Type 2
Pt: Platinum
TGF- β: Transforming growth factor-beta
Fe-Pt: Iron-platinum
ZnO: Zinc oxide
Ni: Nickel
Co–Fe2O4: Cobalt ferrite
Mn–Fe2O4: Manganese Ferrite Nanoparticles
SPIONs: Superparamagnetic iron oxide nanoparticles
AMFs: Alternating magnetic fields
MH: Magnetic hyperthermia
PET: Positron emission tomography
SPECT: Single-photon emission computed tomography
CT: Computed tomography
MRI: Magnetic resonance Imaging
FET: Field-Effect Transistor
Fe3O4: Iron (II,III) oxide
DNA: Deoxyribonucleic acid
LSPR: Localized surface plasmon resonance
H2O2: Hydrogen peroxide
SERS: Surface Enhanced Raman Scattering
DPR: Dipole Plasmon Resonance
PDT: Photodynamic therapy
PTT: Photothermal therapy
TiO_2_: Titanium Dioxide
ROS: Reactive oxygen species
NIR: Near-infrared
AgNPs: Silver nanoparticles
EPR: Enhanced permeability and retention
PEG: Polyethylene glycol
FDA: Food and Drug Administration
EMA: European Medicines Agency
ISO: International Organization for Standardization
AI: Artificial Intelligence
ML: Machine Learning
MNP: Metal nanoparticles
Au: Gold
Ag: Silver
Fe: Iron
Cu: Copper
Pd MNPs: Palladium Metal nanoparticles

## References

[B1] Abbasi N. Ghaneialvar H. Moradi R. Zangeneh M. M. Zangeneh A. (2021). Formulation and characterization of a novel cutaneous wound healing ointment by silver nanoparticles containing citrus lemon leaf: a chemobiological study. Arabian J. Chem. 14, 103246. 10.1016/j.arabjc.2021.103246

[B2] Abbas S. (2021). Green synthesis, characterization and *in-vitro* bioactivities of gold nanoparticles mediated by turmeric crude extract and curcumin. Univ. Tun Hussein Onn Malays. Available online at: https://ir.upm.edu.my/find/Record/my-uthm-ep.3933 .

[B3] Abdal Dayem A. Lee S. B. Cho S.-G. (2018). The impact of metallic nanoparticles on stem cell proliferation and differentiation. Nanomaterials 8, 761. 10.3390/nano8100761 30261637 PMC6215285

[B4] Abuzeid H. M. Julien C. M. Zhu L. Hashem A. M. (2023). Green synthesis of nanoparticles and their energy storage, environmental, and biomedical applications. Crystals 13, 1576. 10.3390/cryst13111576

[B5] Ahmad F. Ashraf N. Ashraf T. Zhou R.-B. Yin D.-C. (2019a). Biological synthesis of metallic nanoparticles (MNPs) by plants and microbes: their cellular uptake, biocompatibility, and biomedical applications. Appl. Microbiol. Biotechnol. 103, 2913–2935. 10.1007/s00253-019-09675-5 30778643

[B6] Ahmad S. Munir S. Zeb N. Ullah A. Khan B. Ali J. (2019b). Green nanotechnology: a review on green synthesis of silver nanoparticles—An ecofriendly approach. Int. J. nanomedicine Vol. 14, 5087–5107. 10.2147/ijn.s200254 PMC6636611 31371949

[B7] Ahmed S. Ahmad M. Swami B. L. Ikram S. (2016a). Green synthesis of silver nanoparticles using Azadirachta indica aqueous leaf extract. J. Radiat. Res. Appl. Sci. 9, 1–7. 10.1016/j.jrras.2015.06.006

[B8] Ahmed S. Ahmad M. Swami B. L. Ikram S. (2016b). A review on plants extract mediated synthesis of silver nanoparticles for antimicrobial applications: a green expertise. J. Adv. Res. 7, 17–28. 10.1016/j.jare.2015.02.007 26843966 PMC4703479

[B9] Ahovan Z. A. Esmaeili Z. Eftekhari B. S. Khosravimelal S. Alehosseini M. Orive G. (2022). Antibacterial smart hydrogels: new hope for infectious wound management. Mater. Today Bio 17, 100499. 10.1016/j.mtbio.2022.100499 PMC9709163 36466959

[B10] Aigbe U. O. Osibote O. A. (2024). Green synthesis of metal oxide nanoparticles, and their various applications. J. Hazard. Mater. Adv. 13, 100401. 10.1016/j.hazadv.2024.100401

[B11] Alabdallah N. M. Hasan M. M. (2021). Plant-based green synthesis of silver nanoparticles and its effective role in abiotic stress tolerance in crop plants. Saudi J. Biol. Sci. 28, 5631–5639. 10.1016/j.sjbs.2021.05.081 34588874 PMC8459083

[B12] Ali A. Aasim M. Çelik K. Nadeem M. A. Baloch F. S. (2024). Frontiers in bacterial-based green synthesized nanoparticles (Nps): a sustainable strategy for combating infectious plant pathogens. Biocatal. Agric. Biotechnol. 60, 103293. 10.1016/j.bcab.2024.103293

[B13] Alibakhshi A. Kahaki F. A. Ahangarzadeh S. Yaghoobi H. Yarian F. Arezumand R. (2017). Targeted cancer therapy through antibody fragments-decorated nanomedicines. J. Control. Release 268, 323–334. 10.1016/j.jconrel.2017.10.036 29107128

[B14] AL-Khattaf F. S. (2021). Gold and silver nanoparticles: green synthesis, microbes, mechanism, factors, plant disease management and environmental risks. Saudi J. Biol. Sci. 28, 3624–3631. 10.1016/j.sjbs.2021.03.078 34121906 PMC8176005

[B15] Allison R. R. Bagnato V. S. Cuenca R. Downie G. H. Sibata C. H. (2006). The future of photodynamic therapy in oncology. Future Oncol. 2, 53–71. 10.2217/14796694.2.1.53 16556073

[B16] AL-Saggaf M. S. (2021). Formulation of insect chitosan stabilized silver nanoparticles with propolis extract as potent antimicrobial and wound healing composites. Int. J. Polym. Sci. 2021, 1–9. 10.1155/2021/5578032

[B17] Alsaiari N. S. Alzahrani F. M. Amari A. Osman H. Harharah H. N. Elboughdiri N. (2023). Plant and microbial approaches as green methods for the synthesis of nanomaterials: synthesis, applications, and future perspectives. Molecules 28, 463. 10.3390/molecules28010463 36615655 PMC9823860

[B18] Alshaer W. Hillaireau H. Fattal E. (2018). Aptamer-guided nanomedicines for anticancer drug delivery. Adv. drug Deliv. Rev. 134, 122–137. 10.1016/j.addr.2018.09.011 30267743

[B19] Alshameri A. W. Owais M. (2022). Antibacterial and cytotoxic potency of the plant-mediated synthesis of metallic nanoparticles Ag NPs and ZnO NPs: a review. OpenNano 8, 100077. 10.1016/j.onano.2022.100077

[B20] AL-Shmgani H. S. Mohammed W. H. Sulaiman G. M. Saadoon A. H. (2017). Biosynthesis of silver nanoparticles from Catharanthus roseus leaf extract and assessing their antioxidant, antimicrobial, and wound-healing activities. Artif. cells, nanomedicine, Biotechnol. 45, 1234–1240. 10.1080/21691401.2016.1220950 27534756

[B21] Alvandi H. Rajati H. Naseriyeh T. Rahmatabadi S. S. Hosseinzadeh L. Arkan E. (2024). Incorporation of Aloe vera and green synthesized ZnO nanoparticles into the chitosan/PVA nanocomposite hydrogel for wound dressing application. Polym. Bull. 81, 4123–4148. 10.1007/s00289-023-04874-7

[B22] Álvarez-Chimal R. Arenas-Alatorre J. Á. (2023). “Green synthesis of nanoparticles: a biological approach,” in Green chemistry for environmental sustainability-prevention-assurance-sustainability (PAS) approach. London UK: IntechOpen.

[B23] Anil Kumar S. Abyaneh M. K. Gosavi S. Kulkarni S. K. Pasricha R. Ahmad A. (2007). Nitrate reductase-mediated synthesis of silver nanoparticles from AgNO 3. Biotechnol. Lett. 29, 439–445. 10.1007/s10529-006-9256-7 17237973

[B24] Anjum S. Hashim M. Malik S. A. Khan M. Lorenzo J. M. Abbasi B. H. (2021). Recent advances in zinc oxide nanoparticles (znO NPs) for cancer diagnosis, target drug delivery, and treatment. Cancers 13, 4570. 10.3390/cancers13184570 34572797 PMC8468934

[B25] Ankamwar B. Damle C. Ahmad A. Sastry M. (2005). Biosynthesis of gold and silver nanoparticles using emblica officinalis fruit extract, their phase transfer and transmetallation in an organic solution. J. Nanosci. Nanotechnol. 5, 1665–1671. 10.1166/jnn.2005.184 16245525

[B26] Antunes Filho S. Dos Santos M. S. Dos Santos O. A. L. Backx B. P. Soran M.-L. Opriş O. (2023). Biosynthesis of nanoparticles using plant extracts and essential oils. Molecules 28, 3060. 10.3390/molecules28073060 37049821 PMC10095647

[B27] Arora P. Sindhu A. Dilbaghi N. Chaudhury A. (2011). Biosensors as innovative tools for the detection of food borne pathogens. Biosens. Bioelectron. 28, 1–12. 10.1016/j.bios.2011.06.002 21763122

[B28] Atala A. Irvine D. J. Moses M. Shaunak S. (2010). Wound healing *versus* regeneration: role of the tissue environment in regenerative medicine. MRS Bull. 35, 597–606. 10.1557/mrs2010.528 PMC3826556 24241586

[B29] Bahrulolum H. Nooraei S. Javanshir N. Tarrahimofrad H. Mirbagheri V. S. Easton A. J. (2021). Green synthesis of metal nanoparticles using microorganisms and their application in the agrifood sector. J. Nanobiotechnology 19, 86–26. 10.1186/s12951-021-00834-3 33771172 PMC7995756

[B30] Baig N. Kammakakam I. Falath W. (2021). Nanomaterials: a review of synthesis methods, properties, recent progress, and challenges. Mater. Adv. 2, 1821–1871. 10.1039/d0ma00807a

[B31] Balakumaran M. Ramachandran R. Balashanmugam P. Mukeshkumar D. Kalaichelvan P. (2016). Mycosynthesis of silver and gold nanoparticles: optimization, characterization and antimicrobial activity against human pathogens. Microbiol. Res. 182, 8–20. 10.1016/j.micres.2015.09.009 26686609

[B32] Balasubramanian S. K. Jittiwat J. Manikandan J. Ong C.-N. Yu L. E. Ong W.-Y. (2010). Biodistribution of gold nanoparticles and gene expression changes in the liver and spleen after intravenous administration in rats. Biomaterials 31, 2034–2042. 10.1016/j.biomaterials.2009.11.079 20044133

[B33] Bamidele M. O. Bamikale M. B. Cárdenas-Hernández E. Bamidele M. A. Castillo-Olvera G. Sandoval-Cortes J. (2025). Bioengineering in solid-state fermentation for next sustainable food bioprocessing. Next Sustain. 6, 100105. 10.1016/j.nxsust.2025.100105

[B34] Banerjee K. Ravishankar Rai V. (2018). A review on mycosynthesis, mechanism, and characterization of silver and gold nanoparticles. BioNanoScience 8, 17–31. 10.1007/s12668-017-0437-8

[B35] Barapatre A. Aadil K. R. Jha H. (2016). Synergistic antibacterial and antibiofilm activity of silver nanoparticles biosynthesized by lignin-degrading fungus. Bioresour. Bioprocess. 3, 8–13. 10.1186/s40643-016-0083-y

[B36] Batool M. Khurshid S. Qureshi Z. Daoush W. M. (2021). Adsorption, antimicrobial and wound healing activities of biosynthesised zinc oxide nanoparticles. Chem. Pap. 75, 893–907. 10.1007/s11696-020-01343-7

[B37] Begum S. R. Jayawardana N. U. (2023). Green synthesized metal nanoparticles as an ecofriendly measure for plant growth stimulation and disease resistance. Plant Nano Biol. 3, 100028. 10.1016/j.plana.2023.100028

[B38] Bhainsa K. C. D'Souza S. (2006). Extracellular biosynthesis of silver nanoparticles using the fungus Aspergillus fumigatus. Colloids surfaces B Biointerfaces 47, 160–164. 10.1016/j.colsurfb.2005.11.026 16420977

[B39] Bharadwaj K. K. Rabha B. Pati S. Sarkar T. Choudhury B. K. Barman A. (2021). Green synthesis of gold nanoparticles using plant extracts as beneficial prospect for cancer theranostics. Molecules 26, 6389. 10.3390/molecules26216389 34770796 PMC8586976

[B40] Bhavani K. S. Anusha T. Brahman P. K. (2021). Platinum nanoparticles decorated on graphitic carbon nitride-ZIF-67 composite support: an electrocatalyst for the oxidation of butanol in fuel cell applications. Int. J. Hydrogen Energy 46, 9199–9214. 10.1016/j.ijhydene.2021.01.006

[B41] Brar K. K. Magdouli S. Othmani A. Ghanei J. Narisetty V. Sindhu R. (2022). Green route for recycling of low-cost waste resources for the biosynthesis of nanoparticles (NPs) and nanomaterials (NMs)-A review. Environ. Res. 207, 112202. 10.1016/j.envres.2021.112202 34655607

[B42] Bukhari A. Ijaz I. Gilani E. Nazir A. Zain H. Saeed R. (2021). Green synthesis of metal and metal oxide nanoparticles using different plants’ parts for antimicrobial activity and anticancer activity: a review article. Coatings 11, 1374. 10.3390/coatings11111374

[B43] Buzea C. Pacheco I. I. Robbie K. (2007). Nanomaterials and nanoparticles: sources and toxicity. Biointerphases 2, MR17–MR71. 10.1116/1.2815690 20419892

[B44] Campaña A. L. Saragliadis A. Mikheenko P. Linke D. (2023). Insights into the bacterial synthesis of metal nanoparticles. Front. Nanotechnol. 5, 1216921. 10.3389/fnano.2023.1216921

[B45] Chatterjee K. Sarkar S. Rao K. J. Paria S. (2014). Core/Shell nanoparticles in biomedical applications. Adv. colloid interface Sci. 209, 8–39. 10.1016/j.cis.2013.12.008 24491963

[B46] Chen M. Xia L. Wu C. Wang Z. Ding L. Xie Y. (2024). Microbe-material hybrids for therapeutic applications. Chem. Soc. Rev. 53, 8306–8378. 10.1039/d3cs00655g 39005165

[B47] Chen X. Pan H. Liu H. DU M. (2010). Nonenzymatic glucose sensor based on flower-shaped au@ Pd core–shell nanoparticles–ionic liquids composite film modified glassy carbon electrodes. Electrochimica Acta 56, 636–643. 10.1016/j.electacta.2010.10.001

[B48] Chinnasamy G. Chandrasekharan S. Koh T. W. Bhatnagar S. (2021). Synthesis, characterization, antibacterial and wound healing efficacy of silver nanoparticles from Azadirachta indica. Front. Microbiol. 12, 611560. 10.3389/fmicb.2021.611560 33679635 PMC7932996

[B49] Conde J. Doria G. Baptista P. (2012). Noble metal nanoparticles applications in cancer. J. drug Deliv. 2012, 1–12. 10.1155/2012/751075 PMC3189598 22007307

[B50] Cucci L. M. Satriano C. Marzo T. LA Mendola D. (2021). Angiogenin and copper crossing in wound healing. Int. J. Mol. Sci. 22, 10704. 10.3390/ijms221910704 34639045 PMC8509573

[B51] Cuccurullo V. DI Stasio G. D. Mazzarella G. Cascini G. L. (2018). Microvascular invasion in HCC: the molecular imaging perspective. Contrast Media and Mol. Imaging 2018, 1–10. 10.1155/2018/9487938 PMC6193341 30402046

[B52] Cuong H. N. Pansambal S. Ghotekar S. Oza R. Hai N. T. T. Viet N. M. (2022). New frontiers in the plant extract mediated biosynthesis of copper oxide (CuO) nanoparticles and their potential applications: a review. Environ. Res. 203, 111858. 10.1016/j.envres.2021.111858 34389352

[B53] Dadfar S. M. Roemhild K. Drude N. I. VON Stillfried S. Knüchel R. Kiessling F. (2019). Iron oxide nanoparticles: diagnostic, therapeutic and theranostic applications. Adv. drug Deliv. Rev. 138, 302–325. 10.1016/j.addr.2019.01.005 30639256 PMC7115878

[B54] Daghian S. G. Farahpour M. R. Jafarirad S. (2021). Biological fabrication and electrostatic attractions of new layered silver/talc nanocomposite using Lawsonia inermis L. and its chitosan-capped inorganic/organic hybrid: investigation on acceleration of *Staphylococcus aureus* and *Pseudomonas aeruginosa* infected wound healing. Mater. Sci. Eng. C 128, 112294. 10.1016/j.msec.2021.112294 34474845

[B55] Domenech M. Marrero-Berrios I. Torres-Lugo M. Rinaldi C. (2013). Lysosomal membrane permeabilization by targeted magnetic nanoparticles in alternating magnetic fields. ACS nano 7, 5091–5101. 10.1021/nn4007048 23705969

[B56] Dougherty T. J. Gomer C. J. Henderson B. W. Jori G. Kessel D. Korbelik M. (1998). Photodynamic therapy. J. Natl. Cancer Inst. 90, 889–905. 10.1093/jnci/90.12.889 9637138 PMC4592754

[B57] Dutt Y. Pandey R. P. Dutt M. Gupta A. Vibhuti A. Raj V. S. (2023). Silver nanoparticles phytofabricated through azadirachta indica: anticancer, apoptotic, and wound-healing properties. Antibiotics 12, 121. 10.3390/antibiotics12010121 36671322 PMC9855199

[B58] EL-Bendary M. A. Afifi S. S. Moharam M. E. Abo EL-Ola S. M. Salama A. Omara E. A. (2021). Biosynthesis of silver nanoparticles using isolated bacillus subtilis: characterization, antimicrobial activity, cytotoxicity, and their performance as antimicrobial agent for textile materials. Prep. Biochem. and Biotechnol. 51, 54–68. 10.1080/10826068.2020.1789992 32701049

[B59] EL-Sayed M. A. (2001). Some interesting properties of metals confined in time and nanometer space of different shapes. Accounts Chem. Res. 34, 257–264. 10.1021/ar960016n 11308299

[B60] Endo T. Ikeda D. Kawakami Y. Yanagida Y. Hatsuzawa T. (2010). Fabrication of core-shell structured nanoparticle layer substrate for excitation of localized surface plasmon resonance and its optical response for DNA in aqueous conditions. Anal. Chim. acta 661, 200–205. 10.1016/j.aca.2009.12.022 20113736

[B61] Evans E. R. Bugga P. Asthana V. Drezek R. (2018). Metallic nanoparticles for cancer immunotherapy. Mater. Today 21, 673–685. 10.1016/j.mattod.2017.11.022 PMC6124314 30197553

[B62] Faghani G. Azarniya A. (2024). Emerging nanomaterials for novel wound dressings: from metallic nanoparticles and MXene nanosheets to metal-organic frameworks. Heliyon 10, e39611. 10.1016/j.heliyon.2024.e39611 39524817 PMC11550055

[B63] Faisal S. Jan H. Shah S. A. Shah S. Khan A. Akbar M. T. (2021). Green synthesis of zinc oxide (ZnO) nanoparticles using aqueous fruit extracts of myristica fragrans: their characterizations and biological and environmental applications. ACS omega 6, 9709–9722. 10.1021/acsomega.1c00310 33869951 PMC8047667

[B64] Faraday M. (1996). Experimental relations of gold (and other metals) to light. SPIE Milest. Ser. MS 120, 9–27. 10.1098/rstl.1857.0011

[B65] Ghosh S. Ahmad R. Zeyaullah M. Khare S. K. (2021). Microbial nano-factories: synthesis and biomedical applications. Front. Chem. 9, 626834. 10.3389/fchem.2021.626834 33937188 PMC8085502

[B66] Gil P. R. Parak W. J. (2008). Composite nanoparticles take aim at cancer. ACS nano 2, 2200–2205. 10.1021/nn800716j 19206383

[B67] Gilchrist R. Medal R. Shorey W. D. Hanselman R. C. Parrott J. C. Taylor C. B. (1957). Selective inductive heating of lymph nodes. Ann. Surg. 146, 596–606. 10.1097/00000658-195710000-00007 13470751 PMC1450524

[B68] Giri A. K. Jena B. Biswal B. Pradhan A. K. Arakha M. Acharya S. (2022). Green synthesis and characterization of silver nanoparticles using Eugenia roxburghii DC. Extract and activity against biofilm-producing bacteria. Sci. Rep. 12, 8383. 10.1038/s41598-022-12484-y 35589849 PMC9120126

[B69] Gole A. Jana N. R. Selvan S. T. Ying J. Y. (2008). Langmuir− blodgett thin films of quantum dots: synthesis, surface modification, and fluorescence resonance energy transfer (FRET) studies. Langmuir 24, 8181–8186. 10.1021/la8000224 18590286

[B70] Gonfa Y. H. Gelagle A. A. Hailegnaw B. Kabeto S. A. Workeneh G. A. Tessema F. B. (2023). Optimization, characterization, and biological applications of silver nanoparticles synthesized using essential oil of aerial part of Laggera tomentosa. Sustainability 15, 797. 10.3390/su15010797

[B71] Gong T. Xie J. Liao J. Zhang T. Lin S. Lin Y. (2015). Nanomaterials and bone regeneration. Bone Res. 3, 15029–7. 10.1038/boneres.2015.29 26558141 PMC4639780

[B72] Gowda B. J. Ahmed M. G. Chinnam S. Paul K. Ashrafuzzaman M. Chavali M. (2022). Current trends in bio-waste mediated metal/metal oxide nanoparticles for drug delivery. J. Drug Deliv. Sci. Technol. 71, 103305. 10.1016/j.jddst.2022.103305

[B73] Grasso G. Zane D. Dragone R. (2019). Microbial nanotechnology: challenges and prospects for green biocatalytic synthesis of nanoscale materials for sensoristic and biomedical applications. Nanomaterials 10, 11. 10.3390/nano10010011 31861471 PMC7023511

[B74] Guilbault G. G. (1983). Determination of formaldehyde with an enzyme-coated piezoelectric crystal detector. Anal. Chem. 55, 1682–1684. 10.1021/ac00261a010

[B75] Guilger-Casagrande M. Germano-Costa T. Bilesky-José N. Pasquoto-Stigliani T. Carvalho L. Fraceto L. F. (2021). Influence of the capping of biogenic silver nanoparticles on their toxicity and mechanism of action towards Sclerotinia sclerotiorum. J. Nanobiotechnology 19, 53–18. 10.1186/s12951-021-00797-5 33627148 PMC7903788

[B76] Gulcin İ. Alwasel S. H. (2022). Metal ions, metal chelators and metal chelating assay as antioxidant method. Processes 10, 132. 10.3390/pr10010132

[B77] Guleria A. Sachdeva H. Saini K. Gupta K. Mathur J. (2022). Recent trends and advancements in synthesis and applications of plant‐based green metal nanoparticles: a critical review. Appl. Organomet. Chem. 36, e6778. 10.1002/aoc.6778

[B78] Gupta A. K. Gupta M. (2005). Synthesis and surface engineering of iron oxide nanoparticles for biomedical applications. biomaterials 26, 3995–4021. 10.1016/j.biomaterials.2004.10.012 15626447

[B79] Guskos N. Likodimos V. Glenis S. Maryniak M. Baran M. Szymczak R. (2008). Magnetic properties of γ-Fe2O3/poly (ether-ester) nanocomposites. J. Nanosci. Nanotechnol. 8, 2127–2134. 10.1166/jnn.2008.063 18572623

[B80] Hamed R. Obeid R. Z. Abu-Huwaij R. (2023). Plant mediated-green synthesis of zinc oxide nanoparticles: an insight into biomedical applications. Nanotechnol. Rev. 12, 20230112. 10.1515/ntrev-2023-0112

[B81] Hamer M. Carballo R. Rezzano I. (2010). Polyallylamine-chlorophyllide derivatized gold and silver nanoparticles as optical probes for sensor applications. Sensors Actuators B Chem. 145, 250–253. 10.1016/j.snb.2009.12.010

[B82] Hano C. Abbasi B. H. (2022). Plant-based green synthesis of nanoparticles: production, characterization and applications. Biomolecules. 12, 31. 10.3390/biom12010031 PMC8773616 35053179

[B83] Hayashi S. Ruppin R. (1985). Raman scattering from GaP microcrystals: analysis of the surface phonon peak. J. Phys. C Solid State Phys. 18, 2583–2592. 10.1088/0022-3719/18/12/019

[B84] Heinrich M. Jalil B. Abdel-Tawab M. Echeverria J. Kulić Ž. Mcgaw L. J. (2022). Best practice in the chemical characterisation of extracts used in pharmacological and toxicological Research—The ConPhyMP—guidelines. Front. Pharmacol. 13, 953205. 10.3389/fphar.2022.953205 36176427 PMC9514875

[B85] Hildebrandt B. Wust P. Ahlers O. Dieing A. Sreenivasa G. Kerner T. (2002). The cellular and molecular basis of hyperthermia. Crit. Rev. oncology/hematology 43, 33–56. 10.1016/s1040-8428(01)00179-2 12098606

[B86] Ho D. Sun X. Sun S. (2011). Monodisperse magnetic nanoparticles for theranostic applications. Accounts Chem. Res. 44, 875–882. 10.1021/ar200090c PMC3184307 21661754

[B87] Hoshyar N. Gray S. Han H. Bao G. (2016). The effect of nanoparticle size on *in vivo* pharmacokinetics and cellular interaction. Nanomedicine 11, 673–692. 10.2217/nnm.16.5 27003448 PMC5561790

[B88] Hosseingholian A. Gohari S. Feirahi F. Moammeri F. Mesbahian G. Moghaddam Z. (2023). Recent advances in green synthesized nanoparticles: from production to application. Mater. Today Sustain. 24, 100500. 10.1016/j.mtsust.2023.100500

[B89] Huang H. Delikanli S. Zeng H. Ferkey D. M. Pralle A. (2010). Remote control of ion channels and neurons through magnetic-field heating of nanoparticles. Nat. Nanotechnol. 5, 602–606. 10.1038/nnano.2010.125 20581833

[B90] Huang Y. He S. Cao W. Cai K. Liang X.-J. (2012). Biomedical nanomaterials for imaging-guided cancer therapy. Nanoscale 4, 6135–6149. 10.1039/c2nr31715j 22929990

[B91] Huston M. Debella M. Dibella M. Gupta A. (2021). Green synthesis of nanomaterials. Nanomaterials 11, 2130. 10.3390/nano11082130 34443960 PMC8400177

[B92] Inaba H. Matsuura K. (2019). Peptide nanomaterials designed from natural supramolecular systems. Chem. Rec. 19, 843–858. 10.1002/tcr.201800149 30375148

[B93] Iravani S. (2011). Green synthesis of metal nanoparticles using plants. Green Chem. 13, 2638–2650. 10.1039/c1gc15386b

[B94] Iravani S. (2014). Bacteria in nanoparticle synthesis: current status and future prospects. Int. Sch. Res. notices 2014, 1–18. 10.1155/2014/359316 PMC4897565 27355054

[B95] Iravani S. Korbekandi H. Mirmohammadi S. V. Zolfaghari B. (2014). Synthesis of silver nanoparticles: chemical, physical and biological methods. Res. Pharm. Sci. 9, 385–406. 26339255 PMC4326978

[B96] Jacinto C. Javed Y. Lavorato G. Tarraga W. A. Conde B. I. C. Orozco J. M. (2025). Biotransformation and biological fate of magnetic iron oxide nanoparticles for biomedical research and clinical applications. Nanoscale Adv. 7, 2818–2886. 10.1039/d5na00195a 40255989 PMC12004083

[B97] Jamkhande P. G. Ghule N. W. Bamer A. H. Kalaskar M. G. (2019). Metal nanoparticles synthesis: an overview on methods of preparation, advantages and disadvantages, and applications. J. drug Deliv. Sci. Technol. 53, 101174. 10.1016/j.jddst.2019.101174

[B98] Javed R. Sajjad A. Naz S. Sajjad H. Ao Q. (2022). Significance of capping agents of colloidal nanoparticles from the perspective of drug and gene delivery, bioimaging, and biosensing: an insight. Int. J. Mol. Sci. 23, 10521. 10.3390/ijms231810521 36142435 PMC9505579

[B99] Jeyaraj M. Gurunathan S. Qasim M. Kang M.-H. Kim J.-H. (2019). A comprehensive review on the synthesis, characterization, and biomedical application of platinum nanoparticles. Nanomaterials 9, 1719. 10.3390/nano9121719 31810256 PMC6956027

[B100] Jiang H.-L. Xu Q. (2011). Catalytic hydrolysis of ammonia borane for chemical hydrogen storage. Catal. Today 170, 56–63. 10.1016/j.cattod.2010.09.019

[B101] Jimenez J. Sheparovych R. Pita M. Narvaez Garcia A. Dominguez E. Minko S. (2008). Magneto-induced self-assembling of conductive nanowires for biosensor applications. J. Phys. Chem. C 112, 7337–7344. 10.1021/jp800013n

[B102] Jo H. Ban C. (2016). Aptamer–nanoparticle complexes as powerful diagnostic and therapeutic tools. Exp. and Mol. Med. 48, e230. 10.1038/emm.2016.44 27151454 PMC4910152

[B103] John A. Shaji A. Velayudhannair K. Nidhin M. Krishnamoorthy G. (2021). Anti-bacterial and biocompatibility properties of green synthesized silver nanoparticles using Parkia biglandulosa (fabales: fabaceae) leaf extract. Curr. Res. Green Sustain. Chem. 4, 100112. 10.1016/j.crgsc.2021.100112

[B104] Kamaraj S.-K. Thirumurugan A. Dhanabalan S. S. Verma S. K. Shajahan S. (2024). Sustainable green synthesised nano-dimensional materials for energy and environmental applications. Florida USA: CRC Press, Taylor and Francis Group.

[B105] Kapinusova G. Lopez Marin M. A. Uhlik O. (2023). Reaching unreachables: obstacles and successes of microbial cultivation and their reasons. Front. Microbiol. 14, 1089630. 10.3389/fmicb.2023.1089630 36960281 PMC10027941

[B106] Kaplan Ö. Tosun N. G. Özgür A. Tayhan S. E. Bilgin S. Türkekul İ. (2021). Microwave-assisted green synthesis of silver nanoparticles using crude extracts of boletus edulis and coriolus versicolor: characterization, anticancer, antimicrobial and wound healing activities. J. Drug Deliv. Sci. Technol. 64, 102641. 10.1016/j.jddst.2021.102641

[B107] Karunakaran G. Sudha K. G. Ali S. Cho E.-B. (2023). Biosynthesis of nanoparticles from various biological sources and its biomedical applications. Molecules 28, 4527. 10.3390/molecules28114527 37299004 PMC10254633

[B108] Kaushik A. Singh R. K. Tyagi P. K. (2023). Green synthesized nanoparticle based drug delivery: recent trends and future prospects. Precis. Nanomedicine 6, 1109–1131. 10.33218/001c.89165

[B109] Kaushik M. Niranjan R. Thangam R. Madhan B. Pandiyarasan V. Ramachandran C. (2019). Investigations on the antimicrobial activity and wound healing potential of ZnO nanoparticles. Appl. Surf. Sci. 479, 1169–1177. 10.1016/j.apsusc.2019.02.189

[B110] Kelly K. L. Coronado E. Zhao L. L. Schatz G. C. (2003). The optical properties of metal nanoparticles: the influence of size, shape, and dielectric environment. The *Journal of Physical Chemistry B* 107 (3), 668–677. 10.1021/jp026731y

[B111] Khan F. Shariq M. Asif M. Siddiqui M. A. Malan P. Ahmad F. (2022). Green nanotechnology: plant-mediated nanoparticle synthesis and application. Nanomaterials 12, 673. 10.3390/nano12040673 35215000 PMC8878231

[B112] Khan I. Saeed K. Khan I. (2019). Nanoparticles: properties, applications and toxicities. Arabian J. Chem. 12, 908–931. 10.1016/j.arabjc.2017.05.011

[B113] Khursheed R. Dua K. Vishwas S. Gulati M. Jha N. K. Aldhafeeri G. M. (2022). Biomedical applications of metallic nanoparticles in cancer: current status and future perspectives. Biomed. and Pharmacother. 150, 112951. 10.1016/j.biopha.2022.112951 35447546

[B114] Kiarashi M. Mahamed P. Ghotbi N. Tadayonfard A. Nasiri K. Kazemi P. (2024). Spotlight on therapeutic efficiency of green synthesis metals and their oxide nanoparticles in periodontitis. J. nanobiotechnology 22, 21. 10.1186/s12951-023-02284-5 38183090 PMC10770920

[B115] Kim D. Jeong Y. Y. Jon S. (2010). A drug-loaded aptamer− gold nanoparticle bioconjugate for combined CT imaging and therapy of prostate cancer. ACS nano 4, 3689–3696. 10.1021/nn901877h 20550178

[B116] Kora A. J. Rastogi L. (2018). Peroxidase activity of biogenic platinum nanoparticles: a colorimetric probe towards selective detection of mercuric ions in water samples. Sensors Actuators B Chem. 254, 690–700. 10.1016/j.snb.2017.07.108

[B117] Kulkarni D. Sherkar R. Shirsathe C. Sonwane R. Varpe N. Shelke S. (2023). Biofabrication of nanoparticles: sources, synthesis, and biomedical applications. Front. Bioeng. Biotechnol. 11, 1159193. 10.3389/fbioe.2023.1159193 37200842 PMC10185809

[B118] Kumar A. Choudhary A. Kaur H. Guha S. Mehta S. Husen A. (2022). Potential applications of engineered nanoparticles in plant disease management: a critical update. Chemosphere 295, 133798. 10.1016/j.chemosphere.2022.133798 35122813

[B119] Kurul F. Turkmen H. Cetin A. E. Topkaya S. N. (2025). Nanomedicine: how nanomaterials are transforming drug delivery, bio-imaging, and diagnosis. Next Nanotechnol. 7, 100129. 10.1016/j.nxnano.2024.100129

[B120] Kyriakides T. R. Raj A. Tseng T. H. Xiao H. Nguyen R. Mohammed F. S. (2021). Biocompatibility of nanomaterials and their immunological properties. Biomed. Mater. 16, 042005. 10.1088/1748-605x/abe5fa PMC8357854 33578402

[B121] Lahiri D. Nag M. Sheikh H. I. Sarkar T. Edinur H. A. Pati S. (2021). Microbiologically-synthesized nanoparticles and their role in silencing the biofilm signaling Cascade. Front. Microbiol. 12, 636588. 10.3389/fmicb.2021.636588 33717030 PMC7947885

[B122] Lam E. Luong J. H. (2014). Carbon materials as catalyst supports and catalysts in the transformation of biomass to fuels and chemicals. ACS Catal. 4, 3393–3410. 10.1021/cs5008393

[B123] Lara P. Philippot K. (2014). The hydrogenation of nitroarenes mediated by platinum nanoparticles: an overview. Catal. Sci. and Technol. 4, 2445–2465. 10.1039/c4cy00111g

[B124] Lee J.-H. Jang J.-T. Choi J.-S. Moon S. H. Noh S.-H. Kim J.-W. (2011). Exchange-coupled magnetic nanoparticles for efficient heat induction. Nat. Nanotechnol. 6, 418–422. 10.1038/nnano.2011.95 21706024

[B125] Liang C. He J. Cao Y. Liu G. Zhang C. Qi Z. (2023). Advances in the application of mxene nanoparticles in wound healing. J. Biol. Eng. 17, 39. 10.1186/s13036-023-00355-7 37291625 PMC10251591

[B126] Li C. Su Y. Lv X. Zuo Y. Yang X. Wang Y. (2012). Au@ Pd core–shell nanoparticles: a highly active electrocatalyst for amperometric gaseous ethanol sensors. Sensors Actuators B Chem. 171, 1192–1198. 10.1016/j.snb.2012.06.073

[B127] Liu X. L. Fan H. M. Yi J. B. Yang Y. Choo E. S. G. Xue J. M. (2012). Optimization of surface coating on Fe 3 O 4 nanoparticles for high performance magnetic hyperthermia agents. J. Mater. Chem. 22, 8235–8244. 10.1039/c2jm30472d

[B128] Liu X. Lee P. Y. Ho C. M. Lui V. C. Chen Y. Che C. M. (2010). Silver nanoparticles mediate differential responses in keratinocytes and fibroblasts during skin wound healing. ChemMedChem 5, 468–475. 10.1002/cmdc.200900502 20112331

[B129] Liu X. Zhang Y. Wang Y. Zhu W. Li G. Ma X. (2020). Comprehensive understanding of magnetic hyperthermia for improving antitumor therapeutic efficacy. Theranostics 10, 3793–3815. 10.7150/thno.40805 32206123 PMC7069093

[B130] Longmire M. Choyke P. L. Kobayashi H. (2008). Clearance properties of nano-sized particles and molecules as imaging agents: considerations and caveats. Nanomedicine 3, 703–717. 10.2217/17435889.3.5.703 18817471 PMC3407669

[B131] Lou L. Chen H. (2023). Functional modification of gelatin-based biodegradable composite films: a review. Food Addit. and Contam. Part A 40, 928–949. 10.1080/19440049.2023.2222844 37310321

[B132] Luo X. Vidal G. D. Killard A. J. Morrin A. Smyth M. R. (2007). Nanocauliflowers: a nanostructured polyaniline‐modified screen‐printed electrode with a self‐assembled polystyrene template and its application in an amperometric enzyme biosensor. Electroanal. Int. J. Devoted Fundam. Pract. Aspects Electroanal. 19, 876–883. 10.1002/elan.200603791

[B133] Lv Y. Yang Y. Fang J. Zhang H. Peng E. Liu X. (2015). Size dependent magnetic hyperthermia of octahedral Fe 3 O 4 nanoparticles. RSC Adv. 5, 76764–76771. 10.1039/c5ra12558h

[B134] Mahdi Z. S. Talebnia Roshan F. Nikzad M. Ezoji H. (2021). Biosynthesis of zinc oxide nanoparticles using bacteria: a study on the characterization and application for electrochemical determination of bisphenol A. Inorg. Nano-Metal Chem. 51, 1–9. 10.1080/24701556.2020.1835962

[B135] Majoumouo M. S. Sharma J. R. Sibuyi N. R. Tincho M. B. Boyom F. F. Meyer M. (2020). Synthesis of biogenic gold nanoparticles from terminalia mantaly extracts and the evaluation of their *in vitro* cytotoxic effects in cancer cells. Molecules 25, 4469. 10.3390/molecules25194469 33003351 PMC7582329

[B136] Malhotra S. P. K. Alghuthaymi M. A. (2022). “Biomolecule-assisted biogenic synthesis of metallic nanoparticles,” in Agri-waste and microbes for production of sustainable nanomaterials, 139–163.

[B137] Malik K. Kazmi A. Sultana T. Raja N. I. Bibi Y. Abbas M. (2025). A mechanistic overview on green assisted formulation of nanocomposites and their multifunctional role in biomedical applications. Heliyon 11, e41654. 10.1016/j.heliyon.2025.e41654 39916856 PMC11800088

[B138] Markus J. Mathiyalagan R. Kim Y.-J. Abbai R. Singh P. Ahn S. (2016). Intracellular synthesis of gold nanoparticles with antioxidant activity by probiotic Lactobacillus kimchicus DCY51T isolated from Korean kimchi. Enzyme Microb. Technol. 95, 85–93. 10.1016/j.enzmictec.2016.08.018 27866630

[B139] Maťátková O. Michailidu J. Miškovská A. Kolouchová I. Masák J. Čejková A. (2022). Antimicrobial properties and applications of metal nanoparticles biosynthesized by green methods. Biotechnol. Adv. 58, 107905. 10.1016/j.biotechadv.2022.107905 35031394

[B140] Mcnamara K. Tofail S. A. (2017). Nanoparticles in biomedical applications. Adv. Phys. X 2, 54–88. 10.1080/23746149.2016.1254570 26024211

[B141] Mehdaoui B. Meffre A. Carrey J. Lachaize S. Lacroix L. M. Gougeon M. (2011). Optimal size of nanoparticles for magnetic hyperthermia: a combined theoretical and experimental study. Adv. Funct. Mater. 21, 4573–4581. 10.1002/adfm.201101243

[B142] Mittal A. K. Chisti Y. Banerjee U. C. (2013). Synthesis of metallic nanoparticles using plant extracts. Biotechnol. Adv. 31, 346–356. 10.1016/j.biotechadv.2013.01.003 23318667

[B143] Mohd Yusof H. Mohamad R. Zaidan U. H. Abdul Rahman N. A. (2019). Microbial synthesis of zinc oxide nanoparticles and their potential application as an antimicrobial agent and a feed supplement in animal industry: a review. J. animal Sci. Biotechnol. 10, 57–22. 10.1186/s40104-019-0368-z PMC6615095 31321032

[B144] Moradifar F. Sepahdoost N. Tavakoli P. Mirzapoor A. (2025). Multi-functional dressings for recovery and screenable treatment of wounds: a review. Heliyon 11, e41465. 10.1016/j.heliyon.2024.e41465 39831167 PMC11742314

[B145] Morsi M. Abdelrazek E. Ramadan R. Elashmawi I. Rajeh A. (2022). Structural, optical, mechanical, and dielectric properties studies of carboxymethyl cellulose/polyacrylamide/lithium titanate nanocomposites films as an application in energy storage devices. Polym. Test. 114, 107705. 10.1016/j.polymertesting.2022.107705

[B146] Mule R. (2024). Synthesis, characterization, and therapeutic potential of biogenic silver nanoparticles using ashwagandha extract. J. Intern. Med. Pharmacol. (JIMP) 1, 66–75. 10.61920/jimp.v1i02.28

[B147] Mulla J. (2024). Nano-based drug delivery systems: a comprehensive review of design, mechanisms and applications. World J. Pharm. 1, 1–11. Available online at: https://www.researchgate.net/publication/391777805.

[B148] Mustapha T. Misni N. Ithnin N. R. Daskum A. M. Unyah N. Z. (2022). A review on plants and microorganisms mediated synthesis of silver nanoparticles, role of plants metabolites and applications. Int. J. Environ. Res. Public Health 19, 674. 10.3390/ijerph19020674 35055505 PMC8775445

[B149] Nandhini S. N. Sisubalan N. Vijayan A. Karthikeyan C. Gnanaraj M. Gideon D. A. M. (2023). Recent advances in green synthesized nanoparticles for bactericidal and wound healing applications. Heliyon 9, e13128. 10.1016/j.heliyon.2023.e13128 36747553 PMC9898667

[B150] Naraginti S. Kumari P. L. Das R. K. Sivakumar A. Patil S. H. Andhalkar V. V. (2016). Amelioration of excision wounds by topical application of green synthesized, formulated silver and gold nanoparticles in albino wistar rats. Mater. Sci. Eng. C 62, 293–300. 10.1016/j.msec.2016.01.069 26952426

[B151] Nasra S. Pramanik S. Oza V. Kansara K. Kumar A. (2024). Advancements in wound management: integrating nanotechnology and smart materials for enhanced therapeutic interventions. Discov. nano 19, 159. 10.1186/s11671-024-04116-3 39354172 PMC11445205

[B152] Nava O. Soto-Robles C. Gómez-Gutiérrez C. Vilchis-Nestor A. Castro-Beltrán A. Olivas A. (2017). Fruit peel extract mediated green synthesis of zinc oxide nanoparticles. J. Mol. Struct. 1147, 1–6. 10.1016/j.molstruc.2017.06.078

[B153] Noman M. Ahmed T. Ijaz U. Hameed A. Shahid M. Azizullah L. I. D. (2023). Microbe-oriented nanoparticles as phytomedicines for plant health management: an emerging paradigm to achieve global food security. Crit. Rev. Food Sci. Nutr. 63, 7489–7509. 10.1080/10408398.2022.2046543 35254111

[B154] Oh I.-H. Min H. S. Li L. Tran T. H. Lee Y.-K. Kwon I. C. (2013). Cancer cell-specific photoactivity of pheophorbide A–Glycol chitosan nanoparticles for photodynamic therapy in tumor-bearing mice. Biomaterials 34, 6454–6463. 10.1016/j.biomaterials.2013.05.017 23755832

[B155] Osman A. I. Zhang Y. Farghali M. Rashwan A. K. Eltaweil A. S. Abd EL-Monaem E. M. (2024). Synthesis of green nanoparticles for energy, biomedical, environmental, agricultural, and food applications: a review. Environ. Chem. Lett. 22, 841–887. 10.1007/s10311-023-01682-3

[B156] Pangli H. Vatanpour S. Hortamani S. Jalili R. Ghahary A. (2021). Incorporation of silver nanoparticles in hydrogel matrices for controlling wound infection. J. Burn Care and Res. 42, 785–793. 10.1093/jbcr/iraa205 33313805 PMC8335948

[B157] Parmar M. Sanyal M. (2022). Extensive study on plant mediated green synthesis of metal nanoparticles and their application for degradation of cationic and anionic dyes. Environ. Nanotechnol. Monit. and Manag. 17, 100624. 10.1016/j.enmm.2021.100624

[B158] Pérez-Lorenzo M. (2012). Palladium nanoparticles as efficient catalysts for suzuki cross-coupling reactions. J. Phys. Chem. Lett. 3, 167–174. 10.1021/jz2013984

[B159] Pita M. Abad J. M. Vaz-Dominguez C. Briones C. Mateo-Martí E. Martín-Gago J. A. (2008). Synthesis of cobalt ferrite core/metallic shell nanoparticles for the development of a specific PNA/DNA biosensor. J. colloid interface Sci. 321, 484–492. 10.1016/j.jcis.2008.02.010 18329659

[B160] Pourmadadi M. Holghoomi R. Maleki-Baladi R. Rahdar A. Pandey S. (2024). Copper nanoparticles from chemical, physical, and green synthesis to medicinal application: a review. Plant Nano Biol. 8, 100070. 10.1016/j.plana.2024.100070

[B161] Priyadarshini E. Priyadarshini S. S. Cousins B. G. Pradhan N. (2021). Metal-fungus interaction: review on cellular processes underlying heavy metal detoxification and synthesis of metal nanoparticles. Chemosphere 274, 129976. 10.1016/j.chemosphere.2021.129976 33979913

[B162] Pulingam T. Foroozandeh P. Chuah J.-A. Sudesh K. (2022). Exploring various techniques for the chemical and biological synthesis of polymeric nanoparticles. Nanomaterials 12, 576. 10.3390/nano12030576 35159921 PMC8839423

[B163] Puri A. Mohite P. Maitra S. Subramaniyan V. Kumarasamy V. Uti D. E. (2024). From nature to nanotechnology: the interplay of traditional medicine, green chemistry, and biogenic metallic phytonanoparticles in modern healthcare innovation and sustainability. Biomed. and Pharmacother. 170, 116083. 10.1016/j.biopha.2023.116083 38163395

[B164] Qiu J. Peng H. Liang R. (2007). Ferrocene-modified Fe3O4@ SiO2 magnetic nanoparticles as building blocks for construction of reagentless enzyme-based biosensors. Electrochem. Commun. 9, 2734–2738. 10.1016/j.elecom.2007.09.009

[B165] Quader S. Kataoka K. (2017). Nanomaterial-enabled cancer therapy. Mol. Ther. 25, 1501–1513. 10.1016/j.ymthe.2017.04.026 28532763 PMC5498831

[B166] Radulescu D.-M. Surdu V.-A. Ficai A. Ficai D. Grumezescu A.-M. Andronescu E. (2023). Green synthesis of metal and metal oxide nanoparticles: a review of the principles and biomedical applications. Int. J. Mol. Sci. 24, 15397. 10.3390/ijms242015397 37895077 PMC10607471

[B167] Radzig M. Nadtochenko V. Koksharova O. Kiwi J. Lipasova V. Khmel I. (2013). Antibacterial effects of silver nanoparticles on gram-negative bacteria: influence on the growth and biofilms formation, mechanisms of action. Colloids Surfaces B Biointerfaces 102, 300–306. 10.1016/j.colsurfb.2012.07.039 23006569

[B168] Rajendran N. K. Kumar S. S. D. Houreld N. N. Abrahamse H. (2018). A review on nanoparticle based treatment for wound healing. J. Drug Deliv. Sci. Technol. 44, 421–430. 10.1016/j.jddst.2018.01.009

[B169] Rami M. R. Meskini M. Sharafabad B. E. (2024). Fungal-mediated nanoparticles for industrial applications: synthesis and mechanism of action. J. Infect. Public Health 17, 102536. 10.1016/j.jiph.2024.102536 39276432

[B170] Saif S. Tahir A. Chen Y. (2016). Green synthesis of iron nanoparticles and their environmental applications and implications. Nanomaterials 6, 209. 10.3390/nano6110209 28335338 PMC5245755

[B171] Salem S. S. Fouda A. (2021). Green synthesis of metallic nanoparticles and their prospective biotechnological applications: an overview. Biol. trace Elem. Res. 199, 344–370. 10.1007/s12011-020-02138-3 32377944

[B172] Sampath S. Madhavan Y. Muralidharan M. Sunderam V. Lawrance A. V. Muthupandian S. (2022). A review on algal mediated synthesis of metal and metal oxide nanoparticles and their emerging biomedical potential. J. Biotechnol. 360, 92–109. 10.1016/j.jbiotec.2022.10.009 36272578

[B173] Samuel M. S. Ravikumar M. John J. A. Selvarajan E. Patel H. Chander P. S. (2022). A review on green synthesis of nanoparticles and their diverse biomedical and environmental applications. Catalysts 12, 459. 10.3390/catal12050459

[B174] Sangeetha G. Rajeshwari S. Venckatesh R. (2011). Green synthesis of zinc oxide nanoparticles by Aloe barbadensis miller leaf extract: structure and optical properties. Mater. Res. Bull. 46, 2560–2566. 10.1016/j.materresbull.2011.07.046

[B175] Scala A. Neri G. Micale N. Cordaro M. Piperno A. (2022). State of the art on green route synthesis of gold/silver bimetallic nanoparticles. Molecules 27, 1134. 10.3390/molecules27031134 35164399 PMC8839662

[B176] Scott R. P. Quaggin S. E. (2015). The cell biology of renal filtration. J. cell Biol. 209, 199–210. 10.1083/jcb.201410017 25918223 PMC4411276

[B177] Selvan D. A. Mahendiran D. Kumar R. S. Rahiman A. K. (2018). Garlic, green tea and turmeric extracts-mediated green synthesis of silver nanoparticles: phytochemical, antioxidant and *in vitro* cytotoxicity studies. J. Photochem. Photobiol. b Biol. 180, 243–252. 10.1016/j.jphotobiol.2018.02.014 29476965

[B178] Sepehri N. Iraji A. Yavari A. Asgari M. S. Zamani S. Hosseini S. (2021). The natural-based optimization of kojic acid conjugated to different thio-quinazolinones as potential anti-melanogenesis agents with tyrosinase inhibitory activity. Bioorg. and Med. Chem. 36, 116044. 10.1016/j.bmc.2021.116044 33640246

[B179] Shafey A. M. E. (2020). Green synthesis of metal and metal oxide nanoparticles from plant leaf extracts and their applications: a review. Green Process. Synthesis 9, 304–339. 10.1515/gps-2020-0031

[B180] Shahabadi N. Zendehcheshm S. Khademi F. Rashidi K. Chehri K. Fatahi dehpahni M. (2021). Green synthesis of Chloroxine-conjugated silver nanoflowers: promising antimicrobial activity and *in vivo* cutaneous wound healing effects. J. Environ. Chem. Eng. 9, 105215. 10.1016/j.jece.2021.105215

[B181] Shah M. Fawcett D. Sharma S. Tripathy S. K. Poinern G. E. J. (2015). Green synthesis of metallic nanoparticles *via* biological entities. Materials 8, 7278–7308. 10.3390/ma8115377 28793638 PMC5458933

[B182] Shahzadi S. Fatima S. Shafiq Z. Janjua M. R. S. A. (2025). A review on green synthesis of silver nanoparticles (SNPs) using plant extracts: a multifaceted approach in photocatalysis, environmental remediation, and biomedicine. RSC Adv. 15, 3858–3903. 10.1039/d4ra07519f 39917042 PMC11800103

[B183] Shaikh S. Nazam N. Rizvi S. M. D. Ahmad K. Baig M. H. Lee E. J. (2019). Mechanistic insights into the antimicrobial actions of metallic nanoparticles and their implications for multidrug resistance. Int. J. Mol. Sci. 20, 2468. 10.3390/ijms20102468 31109079 PMC6566786

[B184] Shankhwar N. Verma A. K. Noumani A. Singh T. Rao K. S. Sharma N. R. (2025). Integrating advanced synthesis techniques and AI-driven approaches with nanofiber technology: a state-of-the-art approach to wound care management. Next Nanotechnol. 8, 100147. 10.1016/j.nxnano.2025.100147

[B185] Shenashen M. A. EL‐Safty S. A. Elshehy E. A. (2014). Synthesis, morphological control, and properties of silver nanoparticles in potential applications. Part. and Part. Syst. Charact. 31, 293–316. 10.1002/ppsc.201300181

[B186] Shi L. Zhao W. Yang Z. Subbiah V. Suleria H. A. R. (2022). Extraction and characterization of phenolic compounds and their potential antioxidant activities. Environ. Sci. Pollut. Res. 29, 81112–81129. 10.1007/s11356-022-23337-6 PMC9606084 36201076

[B187] Siddique S. Chow J. C. (2020). Gold nanoparticles for drug delivery and cancer therapy. Appl. Sci. 10, 3824. 10.3390/app10113824

[B188] Sikiru S. Abiodun O. A. Sanusi Y. K. Sikiru Y. A. Soleimani H. Yekeen N. (2022). A comprehensive review on nanotechnology application in wastewater treatment a case study of metal-based using green synthesis. J. Environ. Chem. Eng. 10, 108065. 10.1016/j.jece.2022.108065

[B189] Singh H. Desimone M. F. Pandya S. Jasani S. George N. Adnan M. (2023). Revisiting the green synthesis of nanoparticles: uncovering influences of plant extracts as reducing agents for enhanced synthesis efficiency and its biomedical applications. Int. J. nanomedicine Vol. 18, 4727–4750. 10.2147/ijn.s419369 PMC10444627 37621852

[B190] Singh J. Dutta T. Kim K.-H. Rawat M. Samddar P. Kumar P. (2018). Green’synthesis of metals and their oxide nanoparticles: applications for environmental remediation. J. nanobiotechnology 16, 84–24. 10.1186/s12951-018-0408-4 30373622 PMC6206834

[B191] Singh K. Singhal S. Pahwa S. Sethi V. A. Sharma S. Singh P. (2024). Nanomedicine and drug delivery: a comprehensive review of applications and challenges. Nano-Structures and Nano-Objects 40, 101403. 10.1016/j.nanoso.2024.101403

[B192] Slavin Y. N. Asnis J. Hńfeli U. O. Bach H. (2017). Metal nanoparticles: understanding the mechanisms behind antibacterial activity. J. nanobiotechnology 15, 65–20. 10.1186/s12951-017-0308-z 28974225 PMC5627441

[B193] Smaoui S. Chérif I. Hlima H. B. Khan M. U. Rebezov M. Thiruvengadam M. (2023). Zinc oxide nanoparticles in meat packaging: a systematic review of recent literature. Food Packag. Shelf Life 36, 101045. 10.1016/j.fpsl.2023.101045

[B194] Sonamuthu J. Cai Y. Liu H. Kasim M. S. M. Vasanthakumar V. R. Pandi B. (2020). MMP-9 responsive dipeptide-tempted natural protein hydrogel-based wound dressings for accelerated healing action of infected diabetic wound. Int. J. Biol. Macromol. 153, 1058–1069. 10.1016/j.ijbiomac.2019.10.236 31756486

[B195] Song J. Y. Kim B. S. (2009). Rapid biological synthesis of silver nanoparticles using plant leaf extracts. Bioprocess Biosyst. Eng. 32, 79–84. 10.1007/s00449-008-0224-6 18438688

[B196] Soni V. Raizada P. Singh P. Cuong H. N. Saini A. Saini R. V. (2021). Sustainable and green trends in using plant extracts for the synthesis of biogenic metal nanoparticles toward environmental and pharmaceutical advances: a review. Environ. Res. 202, 111622. 10.1016/j.envres.2021.111622 34245729

[B197] Sudheer S. Bai R. G. Muthoosamy K. Tuvikene R. Gupta V. K. Manickam S. (2022). Biosustainable production of nanoparticles *via* mycogenesis for biotechnological applications: a critical review. Environ. Res. 204, 111963. 10.1016/j.envres.2021.111963 34450157

[B198] Sun T. Zhang Y. S. Pang B. Hyun D. C. Yang M. Xia Y. (2021). “Engineered nanoparticles for drug delivery in cancer therapy,” in Nanomaterials and neoplasms, 31–142. 10.1002/anie.201403036 25294565

[B199] Tagde P. Najda A. Nagpal K. Kulkarni G. T. Shah M. Ullah O. (2022). Nanomedicine-based delivery strategies for breast cancer treatment and management. Int. J. Mol. Sci. 23, 2856. 10.3390/ijms23052856 35269998 PMC8911433

[B200] Tan X.-C. Zhang J.-L. Tan S.-W. Zhao D.-D. Huang Z.-W. Mi Y. (2009). Amperometric hydrogen peroxide biosensor based on immobilization of hemoglobin on a glassy carbon electrode modified with Fe3O4/chitosan core-shell microspheres. Sensors 9, 6185–6199. 10.3390/s90806185 22454579 PMC3312438

[B201] Tang H. Zhang P. Kieft T. L. Ryan S. J. Baker S. M. Wiesmann W. P. (2010). Antibacterial action of a novel functionalized chitosan-arginine against Gram-negative bacteria. Acta Biomater. 6, 2562–2571. 10.1016/j.actbio.2010.01.002 20060936 PMC2874111

[B202] Thakur N. Ghosh J. Pandey S. K. Pabbathi A. Das J. (2022). A comprehensive review on biosynthesis of magnesium oxide nanoparticles, and their antimicrobial, anticancer, antioxidant activities as well as toxicity study. Inorg. Chem. Commun. 146, 110156. 10.1016/j.inoche.2022.110156

[B203] Thanh N. T. Green L. A. (2010). Functionalisation of nanoparticles for biomedical applications. Nano today 5, 213–230. 10.1016/j.nantod.2010.05.003

[B204] Thatyana M. Dube N. P. Kemboi D. Manicum A.-L. E. Mokgalaka-Fleischmann N. S. Tembu J. V. (2023). Advances in phytonanotechnology: a plant-mediated green synthesis of metal nanoparticles using phyllanthus plant extracts and their antimicrobial and anticancer applications. Nanomaterials 13, 2616. 10.3390/nano13192616 37836257 PMC10574544

[B205] Thiruvengadam R. Kondapavuluri B. K. Thangavelu L. Thiruvengadam M. Hatami M. Kim J. H. (2025). Nanoparticle-based strategies with bioactive compounds for targeting oxidative stress in therapeutic interventions: a comprehensive review. Industrial Crops Prod. 227, 120804. 10.1016/j.indcrop.2025.120804

[B206] Thomas N. G. Varghese N. Kalarikkal N. Thomas S. Sreedharan M. George S. S. (2023). “Toxicity evaluation and biocompatibility of nanostructured biomaterials,” in Cytotoxicity-understanding cellular damage and response. IntechOpen.

[B207] Torres Martin DE Rosales R. Tavaré R. Glaria A. Varma G. Protti A. Blower P. J. (2011). 99mTc-bisphosphonate-iron oxide nanoparticle conjugates for dual-modality biomedical imaging. Bioconjugate Chem. 22, 455–465. 10.1021/bc100483k PMC6205601 21338098

[B208] Trivedi R. Upadhyay T. K. Mujahid M. H. Khan F. Pandey P. Sharangi A. B. (2022). Recent advancements in plant-derived nanomaterials research for biomedical applications. Processes 10, 338. 10.3390/pr10020338

[B209] Vankudoth S. Dharavath S. Veera S. Maduru N. Chada R. Chirumamilla P. (2022). Green synthesis, characterization, photoluminescence and biological studies of silver nanoparticles from the leaf extract of *Muntingia calabura* . Biochem. Biophysical Res. Commun. 630, 143–150. 10.1016/j.bbrc.2022.09.054 36155060

[B210] Vanlalveni C. Ralte V. Zohmingliana H. Das S. Anal J. M. H. Lallianrawna S. (2024). A review of microbes mediated biosynthesis of silver nanoparticles and their enhanced antimicrobial activities. Heliyon 10, e32333. 10.1016/j.heliyon.2024.e32333 38947433 PMC11214502

[B211] Varaprasad K. Karthikeyan C. Yallapu M. M. Sadiku R. (2022). The significance of biomacromolecule alginate for the 3D printing of hydrogels for biomedical applications. Int. J. Biol. Macromol. 212, 561–578. 10.1016/j.ijbiomac.2022.05.157 35643157 PMC13459129

[B212] Veeraraghavan V. P. Periadurai N. D. Karunakaran T. Hussain S. Surapaneni K. M. Jiao X. (2021). Green synthesis of silver nanoparticles from aqueous extract of Scutellaria barbata and coating on the cotton fabric for antimicrobial applications and wound healing activity in fibroblast cells (L929). Saudi J. Biol. Sci. 28, 3633–3640. 10.1016/j.sjbs.2021.05.007 34220213 PMC8241602

[B213] Venkataraman S. (2022). Plant molecular pharming and plant-derived compounds towards generation of vaccines and therapeutics against coronaviruses. Vaccines 10, 1805. 10.3390/vaccines10111805 36366313 PMC9699630

[B214] Vijayaraghavan K. Ashokkumar T. (2017). Plant-mediated biosynthesis of metallic nanoparticles: a review of literature, factors affecting synthesis, characterization techniques and applications. J. Environ. Chem. Eng. 5, 4866–4883. 10.1016/j.jece.2017.09.026

[B215] Vijayaram S. Razafindralambo H. Sun Y.-Z. Vasantharaj S. Ghafarifarsani H. Hoseinifar S. H. (2024). Applications of green synthesized metal Nanoparticles—A review. Biol. Trace Elem. Res. 202, 360–386. 10.1007/s12011-023-03645-9 37046039 PMC10097525

[B216] Vilas-Boas V. Carvalho F. Espiña B. (2020). Magnetic hyperthermia for cancer treatment: main parameters affecting the outcome of *in vitro* and *in vivo* studies. Molecules 25, 2874. 10.3390/molecules25122874 32580417 PMC7362219

[B217] Villagrán Z. Anaya-Esparza L. M. Velázquez-Carriles C. A. Silva-Jara J. M. Ruvalcaba-Gómez J. M. Aurora-Vigo E. F. (2024). Plant-based extracts as reducing, capping, and stabilizing agents for the green synthesis of inorganic nanoparticles. Resources 13, 70. 10.3390/resources13060070

[B218] Wafi A. Khan M. M. (2024). Green synthesized ZnO and ZnO-based composites for wound healing applications. Bioprocess Biosyst. Eng. 48, 521–542. 10.1007/s00449-024-03123-z 39739126

[B219] Wahab S. Salman A. Khan Z. Khan S. Krishnaraj C. Yun S.-I. (2023). Metallic nanoparticles: a promising arsenal against antimicrobial Resistance—Unraveling mechanisms and enhancing medication efficacy. Int. J. Mol. Sci. 24, 14897. 10.3390/ijms241914897 37834344 PMC10573543

[B220] Wang X.-J. Wang L.-L. Huang W.-Q. Tang L.-M. Zou B. Chen K.-Q. (2006). A surface optical phonon assisted transition in a semi-infinite superlattice with a cap layer. Semicond. Sci. Technol. 21, 751–757. 10.1088/0268-1242/21/6/007

[B221] Wang A. Yin L. He L. Xia H. Chen F. Zhao M. (2018). An acidic pH/reduction dual-stimuli responsive nanoprobe for enhanced CT imaging of tumours *in vivo* . Nanoscale 10, 20126–20130. 10.1039/c8nr05061a 30376027

[B222] Wang F. Wang R. Pan Y. DU M. Zhao Y. Liu H. (2022). Gelatin/Chitosan films incorporated with curcumin based on photodynamic inactivation technology for antibacterial food packaging. Polymers 14, 1600. 10.3390/polym14081600 35458350 PMC9032248

[B223] Wang H. Chen Y. Wang L. Liu Q. Yang S. Wang C. (2023). Advancing herbal medicine: enhancing product quality and safety through robust quality control practices. Front. Pharmacol. 14, 1265178. 10.3389/fphar.2023.1265178 37818188 PMC10561302

[B224] Wang Z. Qiao R. Tang N. Lu Z. Wang H. Zhang Z. (2017). Active targeting Theranostic iron oxide nanoparticles for MRI and magnetic resonance-guided focused ultrasound ablation of lung cancer. Biomaterials 127, 25–35. 10.1016/j.biomaterials.2017.02.037 28279919 PMC5400286

[B225] Wu C. Zhang T. Ji B. Chou Y. DU X. (2024). Green synthesis of zinc oxide nanoparticles using Aloe vera leaf extract and evaluation of the antimicrobial and antioxidant properties of the ZnO/regenerated cellulose film. Cellulose 31, 4849–4864. 10.1007/s10570-024-05914-9

[B226] Xiang J. Zhu R. Lang S. Yan H. Liu G. Peng B. (2021). Mussel-inspired immobilization of zwitterionic silver nanoparticles toward antibacterial cotton gauze for promoting wound healing. Chem. Eng. J. 409, 128291. 10.1016/j.cej.2020.128291

[B227] Yadav E. Yadav P. Verma A. (2021). Amelioration of full thickness dermal wounds by topical application of biofabricated zinc oxide and iron oxide nano-ointment in albino wistar rats. J. Drug Deliv. Sci. Technol. 66, 102833. 10.1016/j.jddst.2021.102833

[B228] Yang L. Sun H. Liu Y. Hou W. Yang Y. Cai R. (2018). Self‐assembled aptamer‐grafted hyperbranched polymer nanocarrier for targeted and photoresponsive drug delivery. Angew. Chem. 130, 17294–17298. 10.1002/ange.201809753 PMC6442708 30387923

[B229] Ye L. Cao Z. Liu X. Cui Z. Li Z. Liang Y. (2022). Noble metal-based nanomaterials as antibacterial agents. J. Alloys Compd. 904, 164091. 10.1016/j.jallcom.2022.164091

[B230] Yin Z. F. Wu L. Yang H. G. Su Y. H. (2013). Recent progress in biomedical applications of titanium dioxide. Phys. Chem. Chem. Phys. 15, 4844–4858. 10.1039/c3cp43938k 23450160

[B231] Ying S. Guan Z. Ofoegbu P. C. Clubb P. Rico C. He F. (2022). Green synthesis of nanoparticles: current developments and limitations. Environ. Technol. and Innovation 26, 102336. 10.1016/j.eti.2022.102336

[B232] Younis N. EL Semary N. Mohamed M. (2021). Silver nanoparticles green synthesis *via* cyanobacterium phormidium sp.: characterization, wound healing, antioxidant, antibacterial, and anti-inflammatory activities. Eur. Rev. Med. and Pharmacol. Sci. 25, 3083–3096. 10.26355/eurrev_202104_25563 33877672

[B233] Yuan P. Ding X. Yang Y. Y. Xu Q. H. (2018). Metal nanoparticles for diagnosis and therapy of bacterial infection. Adv. Healthc. Mater. 7, 1701392. 10.1002/adhm.201701392 29582578

[B234] Yu X. Trase I. Ren M. Duval K. Guo X. Chen Z. (2016). Design of nanoparticle‐based carriers for targeted drug delivery. J. Nanomater. 2016, 1–15. 10.1155/2016/1087250 PMC4936496 27398083

[B235] Zhang Y. P. Lee S. H. Reddy K. R. Gopalan A. I. Lee K. P. (2007). Synthesis and characterization of core‐shell SiO2 nanoparticles/poly (3‐aminophenylboronic acid) composites. J. Appl. Polym. Sci. 104, 2743–2750. 10.1002/app.25938

[B236] Zhang C. Lingdong S. Zhang Y. Chunhua Y. (2010). Rare Earth upconversion nanophosphors: synthesis, functionalization and application as biolabels and energy transfer donors. J. Rare Earths 28, 807–819. 10.1016/s1002-0721(09)60206-4

[B237] Zhong Y. Meng F. Deng C. Zhong Z. (2014). Ligand-directed active tumor-targeting polymeric nanoparticles for cancer chemotherapy. Biomacromolecules 15, 1955–1969. 10.1021/bm5003009 24798476

[B238] Zhou L. Zhao X. Li M. Yan L. Lu Y. Jiang C. (2021). Antibacterial and wound healing–promoting effect of sponge-like chitosan-loaded silver nanoparticles biosynthesized by iturin. Int. J. Biol. Macromol. 181, 1183–1195. 10.1016/j.ijbiomac.2021.04.119 33892035

[B239] Zhou Y. Dai Z. (2018). New strategies in the design of nanomedicines to oppose uptake by the mononuclear phagocyte system and enhance cancer therapeutic efficacy. Chemistry–An Asian J. 13, 3333–3340. 10.1002/asia.201800149 29441706

[B240] Zhuang J. Gentry R. W. (2011). “Environmental application and risks of nanotechnology: a balanced view,” in Biotechnology and nanotechnology risk assessment: minding and managing the potential threats around Us. Washington, D.C., USA: ACS Publications.

